# Genome-wide rare copy number variation screening in ulcerative colitis identifies potential susceptibility loci

**DOI:** 10.1186/s12881-016-0289-z

**Published:** 2016-04-01

**Authors:** Hamid Reza Saadati, Michael Wittig, Ingo Helbig, Robert Häsler, Carl A. Anderson, Christopher G. Mathew, Limas Kupcinskas, Miles Parkes, Tom Hemming Karlsen, Philip Rosenstiel, Stefan Schreiber, Andre Franke

**Affiliations:** Institute of Clinical Molecular Biology, Christian-Albrechts-University of Kiel, Schittenhelmstr. 12, 24105 Kiel, Germany; Department of Neuropediatrics, University Clinic Schleswig-Holstein, Campus Kiel, Arnold-Heller-Strasse 3, Building 9, 24105 Kiel, Germany; Wellcome Trust Sanger Institute, Wellcome Trust Genome Campus, Hinxton, Cambridge, UK; Department of Medical and Molecular Genetics, King’s College London School of Medicine, London, UK; Institute for Digestive Research, Lithuanian University of Health Sciences, Mickeviciaus 9, Kaunas, LT 44307 Lithuania; Inflammatory Bowel Disease Research Group, Addenbrooke’s Hospital, University of Cambridge, Cambridge, CB2 2QQ UK; Norwegian PSC Research Center, Clinic for Specialized Medicine and Surgery, Oslo University Hospital, Rikshospitalet, 0027 Oslo, Norway; Department of Internal Medicine, University Hospital Schleswig-Holstein, Schittenhelmstraße 12, 24105 Kiel, Germany

**Keywords:** Ulcerative colitis, Copy number variation, Rare variants, SNP array, Case-control association

## Abstract

**Background:**

Ulcerative colitis (UC), a complex polygenic disorder, is one of the main subphenotypes of inflammatory bowel disease. A comprehensive dissection of the genetic etiology of UC needs to assess the contribution of rare genetic variants including copy number variations (CNVs) to disease risk. In this study, we performed a multi-step genome-wide case-control analysis to interrogate the presence of disease-relevant rare copy number variants.

**Methods:**

One thousand one hundred twenty-one German UC patients and 1770 healthy controls were initially screened for rare deletions and duplications employing SNP-array data. Quantitative PCR and high density custom array-CGH were used for validation of identified CNVs and fine mapping. Two main follow-up panels consisted of an independent cohort of 451 cases and 1274 controls, in which CNVs were assayed through quantitative PCR, and a British cohort of 2396 cases versus 4886 controls with CNV genotypes based on array data. Additional sample sets were assessed for targeted and *in silico* replication.

**Results:**

Twenty-four rare copy number variants (14 deletions and 10 duplications), overrepresented in UC patients were identified in the initial screening panel. Follow-up of these CNV regions in four independent case-control series as well as an additional public *in silico* control group (totaling 4439 UC patients and 15,961 healthy controls) revealed three copy number variants enriched in UC patients; a 15.8 kb deletion upstream of *ABCC4* and *CLDN10* at13q32.1 (0.43 % cases, 0.11 % controls), a 119 kb duplication at 7p22.1, overlapping *RNF216*, *ZNF815*, *OCM* and *CCZ1* (0.13 % cases, 0.01 % controls) and a 134 kb large duplication upstream of the *KCNK9* gene at 8q24.3 (0.22 % carriers among cases, 0.03 % carriers among controls). The trend of association with UC was present after the *P*-values were corrected for combining data from different subpopulations. Break-point mapping of the deleted region suggested non-allelic homologous recombination as the mechanism underlying its formation.

**Conclusion:**

Our study presents a pragmatic approach for effective rare CNV screening of SNP-array data sets and implicates the potential contribution of rare structural variants in the pathogenesis of UC.

**Electronic supplementary material:**

The online version of this article (doi:10.1186/s12881-016-0289-z) contains supplementary material, which is available to authorized users.

## Background

Ulcerative colitis (UC) represents one major subphenotype (OMIM 191390) of human inflammatory bowel disease (IBD, OMIM 266600) and is characterized by chronic inflammation of the intestinal mucosa, exhibiting a continuous pattern in the affected tissue. The disease is more frequent in the northern hemisphere with the prevalence rates ranging from 21 to 246 per 100,000 in North America and Europe [1]. Population-based observations of an 8- to 10-fold greater disease risk among first-degree relatives of UC patients have demonstrated that inherited risk factors contributes to the pathogenesis of UC [2]. However the disease is mainly triggered in genetically susceptible individuals by environmental risk factors [3].

The genetic contribution to UC has been mainly interrogated through genome-wide association studies (GWAS) of mostly common single nucleotide polymorphisms (SNP) represented on oligo-nucleotide microarrays (SNP-array) in the last decade. Subsequent meta analyses of these SNP-GWAS data have substantially increased the number of UC susceptibility loci (*n* > 130), nevertheless the identified risk alleles are mostly of low to modest effects (odds ratio <1.5) and explain less than 15 % of the overall variance in UC risk [4, 5]. It has been assumed that genomic structural variations including CNVs are among the factors that potentially account for the bulky missing heritability of complex disease phenotypes [6]. CNVs comprise insertions, duplications and deletions of genomic sequences, ranging in size from less than 100 base pairs (bp) to greater than 1 Mbp and contribute to higher (>100 fold) DNA sequence variation between individual genomes than do SNPs [7, 8]. Common CNVs, with population frequency of *>* 1 %, often exist in multi-copy number states ranging from 0 to 30 copies per diploid genome [9]. Disease relevance of these common variations has also been explored systematically in some common diseases, but the CNV associations found are far less than that for SNPs [10, 11]. In the case of IBD, copy number differences in a complex region encompassing *ß defensin 2* gene cluster [12] as well as a 20 kb deletion upstream of *IRGM* [13] have been reported as associated with Crohn’s disease (CD), another subphenotype of IBD which has many (>110) susceptibility loci shared with UC [5].

Rare CNVs (frequency *<*1 %) that mainly involve larger genomic segments (*>*100 kb) and occur in fewer copy number states (mostly single copy gain/loss) have been linked extensively to in-born or early-onset neurodevelopmental and intellectual disability disorders with severe and (or) syndromic clinical manifestations [10]. These deleterious CNVs, like other aberrant structural rearrangements - generally known as genomic disorders - mostly arise *de-novo*, are highly penetrant and although recurrent but individually transmit in the population through only one or few generations due to the strong negative selection against them. On the other hand, evidences exist of pathogenic rare CNVs with more moderate effect sizes, which contribute individually or collectively to the susceptibility of common disease phenotypes with lower morbidity/mortality like in Autism, Schizophrenia and Epilepsy [14–16]. Despite this, the potential contribution of rare CNVs to the risk of other complex common diseases like UC is still understudied. To examine whether rare genetic alterations in the form of CNVs affect susceptibility to UC, we employed an existing UC data set, which was used in our previous SNP-GWAS study [17]. The applied platform, Genome-wide human SNP array 6.0, consists of about two million (SNP and copy-number) probe sets and enabled detection of CNVs larger than 15 kb [18]. We investigated CNVs genome-wide using an approach that detects enrichment of multiple overlapping rare variants. Rare deletions and duplications overrepresented in UC cases were identified in the discovery panel. Subsequent validations through independent platform as well as further targeted and *in silico* replications were used to verify the CNVs showing the trend of association with UC.

## Methods

### Study cohorts

We recruited five case control sample sets, one as screening (discovery) panel for rare CNVs and four others for follow-up. Here we describe them upon the platform used for CNV genotyping and origin of the samples;

#### Array-sample sets

Initial screening cohort consisted of 1121 German UC patients and 1770 healthy controls, previously used in our SNP-GWAS experiment using the Affymetrix® Genome-wide Human SNP array 6.0 (Affy6.0) and has been described previously [17]. Details about DNA preparation and sample processing are also described in Additional file 1. Additionally the Affy6.0 data sets of two independent cohorts, one Norwegian and one from UK were recruited. The Norwegian study population consisted of 274 clinically well-characterized UC patients and an ethnically and sex-matched group of Norwegian healthy controls (*n* = 282), also described previously [17]. The UK study population was part of the “Welcome Trust case-control consortium 2” used for UC GWAS [19] and contained data sets of 2396 UC cases and 4886 controls after processing and filtrations described in Additional file 1.

#### TaqMan sample sets

Included two disease cohorts originated from Germany and Lithuania which were genotyped for initially selected CNVs through real-time PCR automated by TaqMan CNV assays (see below). The German UC patients consisted of 245 males and 315 females. The German controls consisted of 779 females of age 18 to 81 (average age: 51) and 637 males of age 27 to 75 (average age: 50), obtained through the biobank PopGen (http://www.popgen.de). The Lithuanian study population consisted of 443 UC patients and a control group of 1157 ethnically, age and sex-matched healthy blood donors.

#### Patient recruitment and ethics

Diagnosis of UC was based on the review of the patients’ original medical records including colonoscopies at the recruiting university hospitals. The currently accepted pathophysiological characteristics of UC include exclusive inflammation of the colon, continuity of inflammation, histological evidence for an inflammation limited to the mucosa, absence of granuloma, intestinal tract architectural changes including crypt abscesses, leukocyte aggregates, distortion of crypt architecture and cryptitis, mucosal edema, and infiltration of neutrophils [20]. These clinical parameters were used to document disease activity as colitis activity index (CAI) [21]. For UC patients, Inclusion criteria were CAI ≥ 4 and endoscopically active disease in the sigmoid colon. Written, informed consent was obtained from all study participants and study setup and all protocols were approved by the national and institutional ethical review committees of the participating centers. A more detailed description of the involved centers in sample recruitment and ethical approval is found in Additional file 1.

#### *In silico* control sample sets

Comprised altogether 6724 individuals recruited in previous genotyping studies as; 60 unrelated HapMap CEU samples genotyped with the Illumina 1 M Dua SNP array [21], 445 CEU controls genotyped with the Illumina 500 k v3 [22], 283 Caucasian controls genotyped with the Illumina Human Hap 300 and 231 Caucasian controls genotyped with the Illumina Human 610-Quad BeadChip [23], 653 Caucasian controls genotyped with the Illumina Human Hap 300 and 551 Caucasian controls genotyped with the Illumina Human 610-Quad BeadChip [24, 25], 3181 European controls genotyped with the Affymetrix® Human SNP array 6.0 [26]. Additional file 1: Table S2 provides the detailed probe coverage of these different platforms for the three specific genomic loci (CNVs) that we evaluated in these samples.

### CNV calling of SNP array datasets

Raw image files were converted into CEL-files by Affymetrix® genotyping console, which were then processed with the Affymetrix Power Tools (APT) apt-copynumber-workflow v 1.67. The values for contrastQC (based on Affymetrix® GTC 3.0.1 User Manual) and MAPD were extracted and samples that failed default QC values were discarded (MAPD > 0.4 and/or contrastQC < 0.4). For the remaining samples an identity by state (IBS) and principal component analysis (PCA) was performed as described previously [17]. The output of apt-copynumber-workflow was used as the input file for our in-house developed CNV data mining tool “CNVineta” [27]. A preliminary batch-wise filtering was performed based on the number of called CNVs per samples. Outliers were defined as samples which had more CNVs than the 75 % quantile plus 1.5 fold of the interquantile range. A rigorous manual raw data inspection for identifying false-negative and -positive CNVs was done subsequently. For the whole data mining process, the predicted CNVs with less than five supporting probes per CNV and mean probe set distance less than one kilobase, were ignored.

#### TaqMan® copy number analysis

Real-time PCR for copy number detection through TaqMan CNV assays was performed as described by Mayo and colleagues [28]. Copy number status of the samples was determined with the software CopyCaller v1.0 from Life Technology (Foster City, CA, USA) for which samples with confirmed and known deletion or duplication were used as calibrator samples (Additional file 1: Figures S3-S5). For technical replication of the CNVs identified during the initial screening, no calibrator sample was included as due to the low frequency of the selected CNVs, we assumed that the majority of samples have a copy number state of two. We discarded samples with confidence values < 95 % and/or z-score ≥ 2.65. At least three of the four technical replicates had to be included for the calculation of confidence values and z-scores.

#### Array CGH (aCGH)

Custom CGH 4 × 72 K Array provided by Nimblegen was used for fine mapping of one deletion and two duplications of interest in 13 individuals. Bed files of the probe design based on NCBI’s build hg18 can be downloaded as Additional file 1. The following regions were covered by the array: chr13:94757799-94817490, chr7:5667022-6057428 and chr8:140281592-140630270.

#### Expression analysis

Endoscopic biopsies from the sigmoid colon of 62 UC patients, of which two individuals carried the relevant deletion, were recruited for gene expression analysis as described previously [21]. TaqMan® pre-designed expression assays were Hs01075312_m1 for *CLDN10* and Hs00988717_m1 for *ABCC4*.

#### Breakpoint mapping

Based on the genomic resolution provided by custom aCGH for the deletion, five flanking primers at each end of the predicted deletion were designed. The subsequent PCR then only yielded amplicons, if a deletion was present (without deletion the fragment is longer than 15 kb, no long range PCR was performed). All possible primer combinations were tested and an amplified fragment of about 610 nucleotides was used for Sanger sequencing. (Forward Primer: 5′-TCCTTCCAGCATATCCCATC; Reverse Primer: 3′- GAATACTGATAACCACAAACAGACAGA). The resulted sequence was then used for BLAT query with the human genome sequence hg18 reference. For the duplications, breakpoints were derived from the aCGH mapping experiment.

## Results

**Fig. 1:**
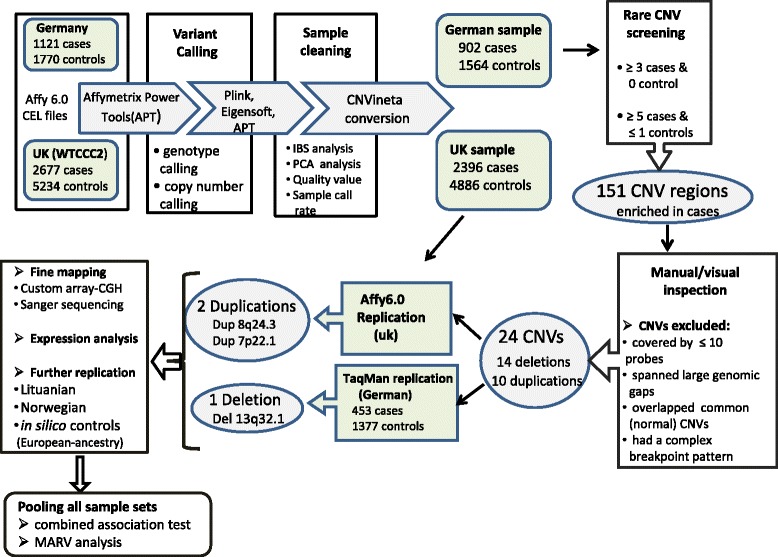
Analysis Workflow. Affymetrix 6.0 data sets for the German (discovery) sample as well as WTCCC2 (UK replication) sample were processed with Affymetrix power tools (APT). Sample cleaning was based on identity by state (IBS) and principal component analysis (PCA) to exclude non-Caucasian samples as well as relatives. The remaining data sets were converted into the CNVineta format. 151 CNVs overrepresented in cases were identified after screening for rare CNVs in the German discovery sample, of which 14 deletion and 10 duplications remained after manual inspection. These 24 CNVs were further evaluated in two independent replication samples, one German and one British (WTCCC2). Dup7p22.1 and Dup8q24.3 were relevant only in UK (Affy6.0) sample, while Del13q32.1 was replicated only in the German (TaqMan) sample. Fine mapping for the deletion was done by Sanger sequencing, while custom array-CGH was used for the two duplications. The status of the 3 relevant CNVs was further evaluated in a Norwegian sample (Affy6.0), a Lithuanian sample (TaqMan) and a control sample of various European individuals from previous published studies. Details of the *in silico* controls (origins, genotyping platform and probe coverage for the three CNV regions) are found in Additional file 1: Table S2. M.A.R.V (Mega Analysis of Rare Variants) approach was used for combining data from different panels

**Fig. 2:**
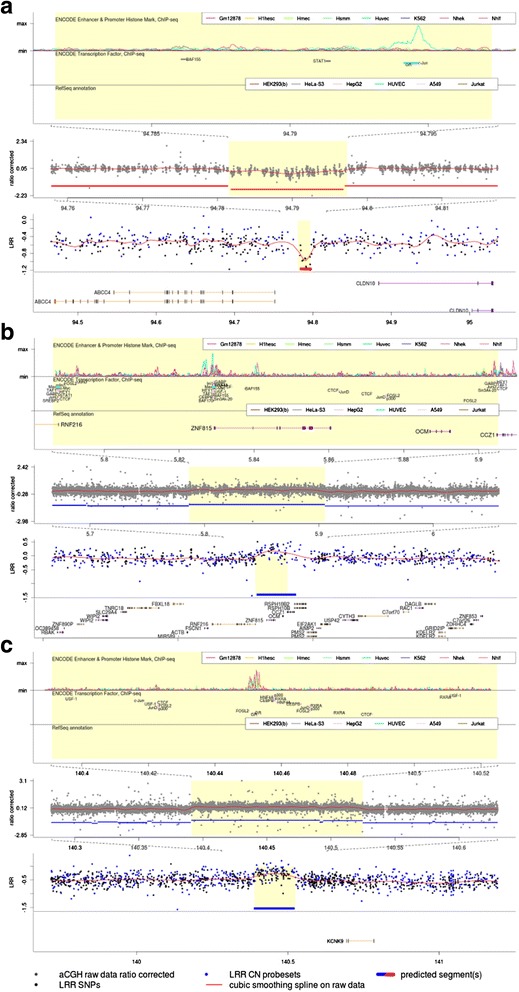
Regional Plots for Del13q32.1 (**a**), Dup7p22.1 (**b**) and Dup8q24.3 (**c**). For each CNV, SNP6.0 array intensity data (lower panel), custom aCGH (middle panel) and ENCODE annotations (upper panel) are visualized. The RefSeq genes are shown in SNP array intensity panel with horizontal orange lines for genes in reverse orientation and purple lines in forward. The red horizontal bar represents the prediction of the deleted segment while the blue bars show duplications. **a** The 15.8 kb deletion at chr13: 94,781,525 - 94,797,285 upstream of *ABCC4* and *CLDN10*. **b** The 119 kb duplication at chr7:5,786,323-5,905,210 encompasses the entire length of the genes *ZNF815* and *OCM*, and partially overlaps *CCZ1* and *RNF216.* **c** The 134 kb large duplication at 8q24.3 (chr8:140,390,975-140,524,875) located upstream of *KCNK9*. An incidence peak (at 140,450 kb) of cis-acting regulatory elements is annotated in the genomic region affected by Duplication

An overall workflow of this study is outlined in Fig. 1. Initially a total of 2891 SNP array 6.0 CEL files (1121 German UC patients/1770 matched controls) were subjected to CNV calling, which then left 2466 samples (902 cases/1564 controls) after discarding outliers with respect to raw data quality, per sample call rate, ethnic origin and relatedness. The primary aim was to identify rare variants in UC patients, which were absent or were underrepresented in controls. Therefore, regions of interest were defined in the primary sample as genomic segments containing CNVs in at least three cases and in no controls. This selection criterion was based on the inspection of the raw data plots where too many false-positives were among the singleton and doubleton predicted events. Furthermore deletions or duplications with one carrier among the controls were also included, when at least five cases contained the corresponding event. Those CNVs occurred in more than two controls were excluded. Upon this setting, the discovery sample yielded 151 CNV regions through screening with our data-mining tool CNVineta. These were then inspected manually and CNV regions were discarded when; predicted event overlapped known common CNVs; had a complex breakpoint pattern; covered by less than 10 probe sets or spanned large genomic gaps (e.g. if a predicted CNV contained a gap which was larger than the part(s) covered by array probes). Twenty-four candidates (14 deletions and 10 duplications) remained after manual inspection of their *Z*-scores, Log *R* ratio and B-allele frequency traces (Additional file 1: Figure S1). The status of these 24 CNVs were then evaluated in two independent cohorts; the WTCCC2 sample (form UK), with CNV genotypes called from Affy6.0 intensity data, and a German cohort (453 UC patients, 1377 controls) genotyped for the selected CNVs by quantitative PCR through TaqMan CNV assays. In British cohort (2394 cases, 4886 controls), of the 24 CNVs evaluated, two duplication events (single copy gains) were the only variants that showed the same distribution trend as the discovery panel, i.e. more represented in cases compared to controls; A 119 kb large duplicated region at 7p22.1 carried by three cases and no control (3/902 cases, 0/1564 controls in discovery panel) and a 134 kb duplication at 8q24.3 with 0.21 % occurrence in cases versus 0.04 % in controls, two-sided Fisher’s exact *P* = 0.058 (*P* = 0.018 in discovery). (Table 1, Additional file 1: Table S1). These two duplications however, were not relevant in the German replication panel, as from the sum of 1830 samples (cases + controls) genotyped for these two variants only one individual (UC case) carried the duplication (Dup8q24.3). Further, of the 24 CNVs followed-up in the German replication panel, a 15.8 kb deletion (single copy loss) at 13q32.1 (chr13: 94,781,525-94,797,285), reproduced the trend of association with nominal *P*-value of 0.005 (*P* = 0.027 in discovery). (Table 1, Additional file 1: Figure S2). Del13q32.1 was not correlated with UC in the WTCCC2 sample. For this deletion and the two aforementioned duplications, we did a validation step, in which the genotypes of all 13 individuals of the discovery panel predicted to carry these 3 CNVs (6 with Del13q32.1, 3 with Dup7p22.1 and 4 with Dup8q24.3) were confirmed through TaqMan assays (Additional file 1: Figure S3). Furthermore we mapped the physical extent of these 3 CNVs more precisely and beyond the resolution of Affy6.0. Figure 2 shows the regional plot of these 3 CNV events, with their breakpoints resolved through custom high density a-CGH. The status of these 3 CNVs was additionally assessed in one small Norwegian sample with affy6.0-based CNV calls as well as a Lithuanian sample (445 cases, 1140 controls) genotyped through corresponding TaqMan CNV assays (Additional file 1: Figures S4–S6). The combined study-wide Fisher’s exact test *P*-value for deletion at 13q32.1 was 1.2 × 10^−3^ (*OR* = 2.64), the duplication at 7p22.1 had a *P*-value of 2.7 × 10^−3^ (*OR* = 8.41) and the duplication at 8q24.3 had a *P*-value of 8.7 × 10^−4^ (*OR* = 4.62). Table 1 lists all panel-wise frequencies as well as combined *P*-values calculated upon “Mega-Analysis of Rare Variants” approach (M.A.R.V) [29] for combining data from different panels of this study.

**Table 1:** Summary of association statistics

Locus:	Deletion 13q32.1						Duplication 7p22.1						Duplication 8q24.3					
Breakpoints:	chr13:94,781,525-94,797,285						chr7:5,786,323-5,905,210						chr8:140,390,975-140,524,875					
Previously reported:	DGV(Variation_49277)						DGV(Variation_53516)											
CNV size:	15.8 kb						119 kb						134 kb					
Covered or close genes:	ABCC4, CLDN10						ZNF815, OCM, RNF216, RSPH10B						KCNK9					
	cases		controls		*P*-value	OR	cases		controls		*P*-value	OR	cases		controls		*P*-value	OR
	wt	cc	wt	cc		(95 % CI)	wt	cc	wt	cc		(95 % CI)	wt	cc	wt	cc		(95 % CI)
Germany (discovery) Affy6.0	897	5	1563	1	0.027	8.71	899	3	1564	0	0.043	Inf	898	4	1564	0	0.018	Inf
						(0.97 - 411)						(0.72 - Inf)	0.45		0.00			(1.15-Inf)
Germany (replication) TaqMan	445	6	1272	2	0.005	8.56	453	0	1279	0	>0.05		452	1	1377	0	>0.05	
						(1.52-87.2)												
WTCCC2 (UK) Affy6.0	2391	5	4880	6	>0.05		2393	3	4886	0	0.061		2391	5	4884	2	0.058	3.40
																		(0.66 - 21.9)
Norwegian Affy6.0	251	1	272	0	0.36		252	0	272	0	>0.05		252	0	272	0	>0.05	
Lithuanian TaqMan	442	2	1131	2	>0.05		438	0	1139	1	>0.05		445	0	1134	0	>0.05	
in silico controls European-ancestry combined results	–	–	4501	5	----		–	–	5788	1	-----		–	–	6724	3	-----	
	4426	19	13619	16	1.2 × 10 ^−3^	2.64	4435	6	14928	2	2.7 × 10 ^−3^	8.41	4438	10	15955	5	8.7 × 10 ^- 4^	4.62
	0.43 %		0.11 %			(1.3–5.2)	0.13 %		0.01 %			(1.4–88)	0.22 %		0.03 %			(1.5–15)
Bonferroni corrected					3.6 × 10 ^−3^						8.1 × 10 ^−3^						2.6 × 10 ^−3^	
MARV					4.3 × 10 ^−3^						6.2 × 10 ^−3^						2.8 × 10 ^−3^	

Frequencies are presented panel-wise and combined for the 3 relevant CNVs. *P*-values were calculated by two-sided Fisher’s exact tests for CNV carriership. Odds ratios (OR) with 95 % confidence intervals (95 % CI; inf = infinite) are listed when *P*-values are smaller than 0.05. cc refers to CNV carrier individuals and wt (wildtype) to non-carriers. To account for population structure and low frequencies, the M.A.R.V. analysis method [29] was applied to the overall study sample

For Del13q32.1, fine mapping through high density a-CGH followed by Sanger sequencing identified the sequence motif 5′-GATCAC-3′ at both breakpoints of the deleted segment. As the deletion is flanked by 16 highly identical *Alu* repeats, it is very likely that non-allelic homologous recombination (NAHR) is the underlying mechanism of the event [30]. We further analyzed the expression levels of the two nearby genes of this deletion, namely *ABCC4* and *CLDN10* in inflamed intestinal biopsies of two patients harboring the deletion and 60 UC patients without it (Additional file 1: Figure S7). Intriguingly, the *CLDN10* expression was significantly lower in both deletion-carrier patients in comparison with the median expression of non-carriers within inflamed mucosa.

## Discussion

Employing our existing UC-GWAS data set for CNV calling coupled with our in-house developed CNV data-mining tool, we performed a genome-wide scan for rare CNVs associated with UC. After follow-up genotyping in four independent case control samples we identified three rare candidate CNVs, one deletion and two duplications as overrepresented (at a nominal significance) in UC patients, compared to controls. Del13q32.1, as a 15.8 kb single copy loss at chr13: 94,781,525-94,797,285 showed the trend of association in the discovery panel, which was reproduced in the so called TaqMan replication panel originated from Germany. However, correlation of Del13q32.1with the disease phenotype was not observed in the WTCCC2-UC panel. The two duplications i.e. Dup7p22.1 (chr7:5,786,323-5,905,210) and Dup8q24.3 (chr8:140,390,975-140,524,875) were overrepresented in UC patients of the discovery panel (compared to controls) and this trend was replicated in the WTCCC2 cohort, although no association was seen for these duplications in two independent replication panels with German and Lithuanian origins. We further evaluated the status of these three CNVs in an *in silico* data set comprising a total of 6727 unrelated control individuals of European ancestry. The scarce occurrence of the three variants (5 of 4505 for Del13q32.1, 1 of 5788 for Dup7p22.1 and 3 of 6727 for Dup8q24.3), observed in these samples was consistent with their low frequencies in our discovery and replication panels.

It should be mentioned that CNV discovery platform used here (Affy6.0), although having a high probe density, has the limited resolution of detecting CNVs that are larger than ~15 kb [9]. Therefore, possible smaller CNV events (<15 kb), probably of disease relevance, have not been examined in our study. On the other hand in comparison to the small median size of common CNVs (~3 kb) in the human genome [8, 10], the three rare CNVs identified here, are intermediate to large genomic alterations, involving regions with multiple genes, which can potentially result in functional (deleterious) consequences leading to disease pathogenesis.

Dup7p21.1 indeed lies in a very gene rich region, encompassing either their entire lengths (ZNF815, OCM) or overlapping (RNF216, RSPH10B) partially. Dup8q24.3 is a 134 kb large duplication upstream of the gene *KCNK9* (*TASK3)*, which encodes a member of the subfamily K of the potassium channel proteins. The ever-increasing knowledge about the involvement of *TASK3 (TWIK-related acid-sensitive potassium)* channels in the pathogenesis of autoimmune inflammation [31, 32] have converted them from “mere background” channels to key modulators in pathophysiological conditions.

Del13q32.1 is located 33.7 kb upstream of the gene *ABCC4* (ATP-binding cassette, sub-family C, member 4) also known as *MRP4* (multidrug resistance-associated protein4), and 78.5 kb upstream of *CLDN10* (*claudin 10*). ABCC4/MRP4 belongs to a large family of trans-membrane proteins which play an important role in regulating cAMP-dependent signaling pathways [33] as well as human dendritic cell migration and thereby modulating immune response [34]. Mapping of MRP protein expression among different regions of the human intestinal tract has showed that MRPs are higher expressed in the colon compared to the ileum [35]. Interestingly, one member of this protein family i.e. ABCC1/MRP1 has been previously associated with severe UC but not with CD [36]. The other neighboring gene *CLDN10*, coding for a tight junction adhesion protein is also an intriguing candidate regarding the molecular pathogenesis of UC. Tight junctions contribute essentially to the intestinal epithelial integrity. Barrier disruptions are known to be one of the main hallmarks of both phenotypes (CD and UC) of inflammatory bowel disease and various genes involved in epithelial barrier maintenance have been associated with IBD [37–39]. Moreover, changes in expression and distribution of *Claudin* 2, 5 and 8 have been shown to result in discontinuous tight junctions and barrier dysfunction in active CD [40]. For interrogating the probable effect of the deletion on the expression of the two nearby genes, we examined two intestinal biopsy samples of unrelated patients carrying the deletion. Compared with the *CLDN10* expression level of the deletion-depleted UC patients in inflamed mucosa, the biopsy specimen from the patient with the deletion showed very low level of expression. Yet, this differential expression was not clear for *ABCC4.* Due to the sparsity of the deletion variation, no more biopsies from distinct patients harboring the deletion were available to further verify the effect of deletion on the expression of these two genes. However, presence of cis-acting regulatory elements such as transcription factor binding sites, showed in ENCODE annotations of the deleted region might be an explanation of the distinct *CLDN10* expression we observed here.

Overall we find that the rare CNV candidates of this study, verified by visual inspection of the underlying raw data, are true positive CNVs. All three relevant CNVs could technically be validated by independent methods and were followed-up in independent sample sets. In contrast to common variants, disease correlation of rare variants is difficult to be assessed through classical association statistics. Low frequency of these variants impedes to detect associations at the genome-wide level significance by modest or intermediate sample sizes. Power limitations may even increase when these variants do not have high penetrance. In this study, the trend of association with UC was present for each of the three mentioned copy number variants in the discovery sample as well as in at least one replication panel, nevertheless higher statistical power, provided by larger case control samples are needed to confidently evaluate the disease risk of these variants.

## Conclusion

Our multi-step case control analysis introduced rare structural variants with a potential contribution to the risk of UC. While further follow-up studies in larger disease cohorts as well as functional experimental assays are needed to conclusively verify the disease relevance of these three loci, we showed that existing GWAS data sets may still be of use in extending the knowledge of genetic etiology in common complex diseases.

## Additional file

Additional file 1: Supplementary material. (DOCX 7254 kb)

## References

[1] Loftus EV (2004). Clinical epidemiology of inflammatory bowel disease: incidence, prevalence, and environmental influences. Gastroenterology.

[2] Orholm M, Munkholm P, Langholz E, Nielsen OH, Sorensen TI, Binder V (1991). Familial occurrence of inflammatory bowel disease. N Engl J Med.

[3] Ananthakrishnan AN (2015). Epidemiology and risk factors for IBD. Nat Rev Gastroenterol Hepatol.

[4] Anderson CA, Boucher G, Lees CW, Franke A, D’Amato M, Taylor KD (2011). Meta-analysis identifies 29 additional ulcerative colitis risk loci, increasing the number of confirmed associations to 47. Nat Genet.

[5] Jostins L, Ripke S, Weersma RK, Duerr RH, McGovern DP, Hui KY (2012). Host-microbe interactions have shaped the genetic architecture of inflammatory bowel disease. Nature.

[6] Eichler EE, Flint J, Gibson G, Kong A, Leal SM, Moore JH, Nadeau JH (2010). Missing heritability and strategies for finding the underlying causes of complex disease. Nat Rev Genet.

[7] Conrad DF, Pinto D, Redon R, Feuk L, Gokcumen O, Zhang Y, Aerts J (2010). Origins and functional impact of copy number variation in the human genome. Nature.

[8] McCarroll SA (2010). Copy number variation and human genome maps. Nat Genet.

[9] Alkan C, Coe BP, Eichler EE (2011). Genome structural variation discovery and genotyping. Nat Rev Genet.

[10] Girirajan S, Campbell CD, Eichler EE (2011). Human copy number variation and complex genetic disease. Annu Rev Genet.

[11] Consortium WTCC, Craddock N, Hurles ME, Cardin N, Pearson RD, Plagnol V (2010). Genome-wide association study of CNVs in 16,000 cases of eight common diseases and 3,000 shared controls. Nature.

[12] Fellermann K, Stange DE, Schaeffeler E, Schmalzl H, Wehkamp J, Bevins CL, Reinisch W (2006). A chromosome 8 gene-cluster polymorphism with low human beta-defensin 2 gene copy number predisposes to Crohn disease of the colon. Am J Hum Genet.

[13] McCarroll SA, Huett A, Kuballa P, Chilewski SD, Landry A, Goyette P (2008). Deletion polymorphism upstream of IRGM associated with altered IRGM expression and Crohn’s disease. Nat Genet.

[14] Glessner JT, Wang K, Cai G, Korvatska O, Kim CE, Wood S, Zhang H (2009). Autism genome-wide copy number variation reveals ubiquitin and neuronal genes. Nature.

[15] Stefansson H, Rujescu D, Cichon S, Pietiläinen OP, Ingason A, Steinberg S, Fossdal R (2008). Large recurrent microdeletions associated with schizophrenia. Nature.

[16] Helbig I, Mefford HC, Sharp AJ, Guipponi M, Fichera M, Franke A (2009). 15q13.3 microdeletions increase risk of idiopathic generalized epilepsy. Nat Genet.

[17] Franke A, Balschun T, Sina C, Ellinghaus D, Häsler R, Mayr G, Albrecht M (2010). Genome-wide association study for ulcerative colitis identifies risk loci at 7q22 and 22q13 (IL17REL). Nat Genet.

[18] McCarroll SA, Kuruvilla FG, Korn JM, Cawley S, Nemesh J, Wysoker A (2008). Integrated detection and population-genetic analysis of SNPs and copy number variation. Nat Genet.

[19] Barrett JC, Lee JC, Lees CW, Prescott NJ, Anderson CA, Phillips A, UK IBD Genetics Consortium (2009). Genome-wide association study of ulcerative colitis identifies three new susceptibility loci, including the HNF4A region. Nature Genet.

[20] Podolsky DK (2002). Inflammatory bowel disease. N Engl J Med.

[21] Costello CM, Mah N, Häsler R, Rosenstiel P, Waetzig GH, Hahn A, Lu T, Gurbuz Y (2005). Dissection of the inflammatory bowel disease transcriptome using genome-wide cDNA microarrays. PLoS Med.

[22] Cooper GM, Zerr T, Kidd JM, Eichler EE, Nickerson DA (2008). Systematic assessment of copy number variant detection via genome-wide SNP genotyping. Nat Genet.

[23] Simon-Sanchez J, Scholz S, Fung HC, Matarin M, Hernandez D, Gibbs JR, Britton A, de Vrieze FW (2007). Genome-wide SNP assay reveals structural genomic variation, extended homozygosity and cell-line induced alterations in normal individuals. Hum Mol Genet.

[24] Simon JA, Lin F, Hulley SB, Blanche PJ, Waters D, Shiboski S, Rotter JI, Nickerson DA (2006). Phenotypic predictors of response to simvastatin therapy among African-Americans and Caucasians: the Cholesterol and Pharmacogenetics (CAP) Study. Am J Cardiol.

[25] Albert MA, Danielson E, Rifai N, Ridker PM, PRINCE Investigators (2001). Effect of statin therapy on C-reactive protein levels: the pravastatin inflammation/CRP evaluation (PRINCE): a randomized trial and cohort study. JAMA.

[26] Stone JL, O’Donovan MC, Gurling H, Kirov GK, Blackwood DH, Corvin A, Craddock NJ (2008). Rare chromosomal deletions and duplications increase risk of schizophrenia. Nature.

[27] Wittig M, Helbig I, Schreiber S, Franke A (2010). CNVineta: a data mining tool for large case-control copy number variation datasets. Bioinformatics.

[28] Mayo P, Hartshorne T, Li K, McMunn-Gibson C, Spencer K, Schnetz-Boutaud N (2010). CNV analysis using TaqMan copy number assays. Curr Protoc Hum Genet.

[29] Rivas MA, Beaudoin M, Gardet A, Stevens C, Sharma Y, Zhang CK, Boucher G (2011). Deep resequencing of GWAS loci identifies independent rare variants associated with inflammatory bowel disease. Nat Genet.

[30] Hastings PJ, Lupski JR, Rosenberg SM, Ira G (2009). Mechanisms of change in gene copy number. Nat Rev Genet.

[31] Bittner S, Budde T, Wiendl H, Meuth SG (2010). From the background to the spotlight: TASK channels in pathological conditions. Brain Pathol.

[32] Meuth SG, Bittner S, Meuth P, Simon OJ, Budde T, Wiendl H (2008). TWIK-related acid-sensitive K+ channel 1 (TASK1) and TASK3 critically influence T lymphocyte effector functions. J Biol Chem.

[33] Sassi Y, Lipskaia L, Vandecasteele G, Nikolaev VO, Hatem SN, Cohen Aubart F, Russel FG (2008). Multidrug resistance-associated protein 4 regulates cAMP-dependent signaling pathways and controls human and rat SMC proliferation. J Clin Invest.

[34] van de Ven R, Scheffer GL, Reurs AW, Lindenberg JJ, Oerlemans R, Jansen G, Gillet JP (2008). A role for multidrug resistance protein 4 (MRP4;ABCC4) in human dendritic cell migration. Blood.

[35] Zimmermann C, Gutmann H, Hruz P, Gutzwiller JP, Beglinger C, Drewe J (2005). Mapping of multidrug resistance gene 1 and multidrug resistance-associated protein isoform 1 to 5 mRNA expression along the human intestinal tract. Drug Metab Dispos.

[36] Onnie CM, Fisher SA, Pattni R, Sanderson J, Forbes A, Lewis CM, Mathew CG, et al. Associations of allelic variants of the multidrug resistance gene (ABCB1 or MDR1) and inflammatory bowel disease and their effects on disease behavior: a case-control and meta-analysis study. Inflamm Bowel Dis. 2006;12(4):263–71. 10.1097/01.MIB.0000209791.98866.ba 16633048

[37] Schmitz H, Barmeyer C, Fromm M, Runkel N, Foss HD, Bentzel CJ, Riecken EO, Schulzke JD (1999). Altered tight junction structure contributes to the impaired epithelial barrier function in ulcerative colitis. Gastroenterology.

[38] Berkes J, Viswanathan VK, Savkovic SD, Hecht G (2003). Intestinal epithelial responses to enteric pathogens: effects on the tight junction barrier, ion transport, and inflammation. Gut.

[39] Lees CW, Barrett JC, Parkes M, Satsangi J (2011). New IBD genetics: common pathways with other diseases. Gut.

[40] Zeissig S, Bürgel N, Günzel D, Richter J, Mankertz J, Wahnschaffe U, Kroesen AJ (2007). Changes in expression and distribution of claudin 2, 5 and 8 lead to discontinuous tight junctions and barrier dysfunction in active Crohn’s disease. Gut.

