# Supplementary material for: Genome-wide rare copy number variation screening in ulcerative colitis identifies potential susceptibility loci
Source: BMC Med Genet. 2016 Apr 1;17:26. doi: 10.1186/s12881-016-0289-z (PMC4818401; doi:10.1186/s12881-016-0289-z)
Supplement: Supplementary file 1 — Supplementary material. (DOCX 7254 kb) [file 12881_2016_289_MOESM1_ESM.docx]

**Additional file 1**

**Genome-wide Rare Copy Number Variation Screening in Ulcerative Colitis Identifies Potential Susceptibility Loci**

Hamid Reza Saadati**^1^** , Michael Wittig**^1^**, Ingo Helbig**^2^**, Robert Häsler**^1^**, Carl A. Anderson**^3^**, Christopher G. Mathew**^4^**, Limas Kupcinskas**^5^**, Miles Parkes**^6^**, Philip Rosenstiel**^1^**,Tom Hemming Karlsen**^7^**, Stefan Schreiber**^1,8^** and Andre Franke**^1,‡^**

**^1^** Institute of Clinical Molecular Biology, Christian-Albrechts-University, 24105 Kiel, Germany

**^2^** Department of Neuropediatrics, University Clinic Schleswig-Holstein, Campus Kiel, Arnold-Heller-Strasse 3, Building 9, 24105 Kiel, Germany

^3^ Wellcome Trust Sanger Institute, Wellcome Trust Genome Campus, Hinxton, Cambridge, UK

**^4^** King’s College London School of Medicine, Guy’s Hospital, Department of Medical and Molecular Genetics, London, UK

**^5^** Institute for Digestive Research, Lithuanian University of Health Sciences, Mickeviciaus 9, Kaunas, LT, 44307, Lithuania

**^6^** Inflammatory Bowel Disease Research Group, Addenbrooke's Hospital, University of Cambridge, Cambridge CB2 2QQ, UK **^7^** Norwegian PSC Research Center, Clinic for Specialized Medicine and Surgery, Oslo University Hospital, Rikshospitalet, 0027 Oslo, Norway.

**^8^** Department of internal medicine, University Hospital Schleswig-Holstein, Schittenhelmstraße 12, 24105 Kiel, Germany

**Supplementary Methods**

**Sample Recruitment and Ethics**

German patients of the discovery and replication panels were recruited either at the Department of General Internal Medicine of the Christian-Albrechts-University Kiel, the Charité University Hospital Berlin, through local outpatient services, or nationwide with the support of the German Crohn and Colitis Foundation. Clinical, radiological, histological, and endoscopic (i.e. type and distribution of lesions) examinations were required to unequivocally confirm the diagnosis of ulcerative colitis (UC) [1]. 1214 German healthy control individuals of discovery panel (1703 total) were obtained from the biobank PopGen (<http://www.popgen.de>). The remaining 489 German healthy controls in discovery panel were selected from the KORA F4 survey, an independent population-based sample from the general population living in the region of Augsburg, Southern Germany [2]. Written, informed consent was obtained from all study participants and all protocols were approved either by the ethical committee of the University-Hospital Schleswig-Holstein, Center Kiel or through the institutional committee of the “Kompetenznetz Darmerkrankungen” (<http://www.kompetenznetz-ced.de/>) in Germany.

The 274 clinically well-characterized Norwegian UC patients of replication panel were recruited through a population-based incidence study, the Inflammatory Bowel disease in South-Eastern Norway (IBSEN) study [3]. An ethnically and sex-matched group of Norwegian healthy controls (n=282) was randomly selected from the Norwegian Bone Marrow Donor Registry (NBMDR). The strict criteria (including absence of any autoimmune disease) on inclusion in the NBMDR ensured correct classification of these controls as healthy. Norwegian sample recruitment was approved by the ethics committee of Oslo University Hospital, Rikshospitalet, Norway.

The Lithuanian study population consisted of 443 UC patients recruited at 6 hospitals in Lithuania: Kaunas Medical University Hospital (Kaunas), Vilnius University Hospital Santariskiu Clinic(Vilnius), M. Marcinkevicius Hospital (Vilnius), Klaipeda Seamen’s Hospital (Klaipeda),Panevezys Regional Hospital (Panevezys), Siauliai Regional Hospital (Siauliai) and 2hospitals in Latvia: P. Stradin Clinical University Hospital (Riga) and Riga Eastern Clinical University Hospital, Clinic Linezers (Riga). The diagnosis of UC was based on standard clinical, endoscopic, radiological and histological criteria. The control group consisted of 1157 ethnically, age and sex-matched healthy blood donors. Written, informed consent was obtained from all study participants and all protocols were approved by the institutional ethical review committee of the Lithuanian University of Health Sciences, Kaunas, Lithuania.

**SNP array genotyping**

The genotyping for the German discovery panel and Norwegian panel - which were both part of the German NGFN GWAS initiative (see press release 04-26-07 on http://www.ngfn.de/englisch/index_368.htm) funded by the NGFN – was performed by an Affymetrix^®^ service facility (South San Francisco, CA, USA) using the Affymetrix^®^ Genome- Wide Human SNP Array 6.0 (1000k) (Santa Clara, CA, USA). The array is based on an assay termed whole-genome sampling analysis (WGSA) developed for highly multiplexed SNP genotyping of complex DNA. This method reproducibly amplifies a subset of the human genome through a single primer amplification reaction using restriction enzyme digested, adapter-ligated human genomic DNA. In brief, 5 μl of genomic DNA samples at 50 ng/ul were aliquoted to the corresponding wells of two 96-well plates. The first run of samples was processed as an entire plate. In the lab, transfers were made with a 12-channel pipette, reducing the risk of sample tracking errors. One plate was digested with NspI and the other plate was digested with StyI. The reaction was incubated at 37 °C for 2 hours and at 65 °C for 20 minutes to deactivate the enzyme. The digested DNA was then ligated to their respective NspI adaptor and StyI adaptor. The ligated product was then PCR-amplified using a common primer. Both NspI PCR product and StyI PCR product were combined, and then purified by ethanol precipitation in combination with membrane filter plate. Purified PCR product was further fragmented with DNase I then labeled with biotin. Labeled NA was combined with hybridization mix and then injected into array. Arrays were hybridized for 18 to 22 hours at 50°C. DNA samples were recovered from arrays and washed and stained by using Affymetrix® FS450 fluidic stations. Stained arrays were scanned using Affymetrix® GeneChip Scanner 3000 7G.

**WTCCC2 data processing**

First the dataset was cleaned based on genotype calling. Genotype calling was performed using the Birdseed v2 algorithm implemented in Affymetrix Power Tools version 1.12.0 [4]. Subsequently, genotypes with their corresponding intensity values were converted into the Beagle format by means of python scripts. Because we observed an excess of genotyping artifacts that would result in false positive associations, the software Beaglecall v1.0.1 (Browning and Yu 2009) was used to generate accurate genotype calls by using both allele signal intensities and inter-marker correlation. Beaglecall’s built-in data quality filters excluded any marker with large deviation from the Hardy-Weinberg equilibrium (*P*HWE < 10-6) or with > 5% of samples having maximal genotype probability < 0.95. We also excluded individuals from each pair of unexpected duplicates or relatives, as well as individuals with outlier heterozygosities of ± 5 s.d. away from the mean. The remaining GWAS samples were tested for population stratification using the principal components stratification method, as implemented in EIGENSTRAT [5], and population outliers were subsequently excluded. SNPs that had a minor allele frequency < 1% and exact Hardy-Weinberg equilibrium *P*controls < 10-4 were finally excluded. For the remaining samples a CNV calling was performed. CEL files were processed with the Affymetrix Power Tools (APT) apt-copynumber-workflow v 1.67. The values for contrastQC and MAPD were extracted and samples that failed default QC values were discarded (MAPD > 0.4 and/or contrastQC < 0.4). The apt-copynumber-workflow output was converted to CNVineta format [6]. A preliminary filtering was performed based on the number of called CNVs per samples. This was performed batch wise, as one batch consists of a sample collection which was prepared in the same process. Outlier were defined as samples which had more CNVs than the 75% quantile plus 1.5 fold of the interquantile range.

**Suppl. figure 1. Twenty-four regions with rare CNVs overrepresented in UC cases of the German discovery panel**

For each region the predicted CNVs are shown for cases (upper panel) and controls (lower panel) with Duplications in blue and deletions in red. Involved RefSeq genes are annotated. SNP probe sets in black and copy number probesets in blue.


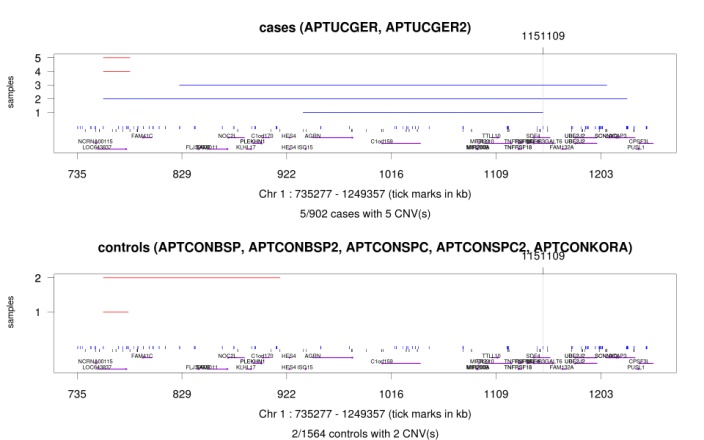

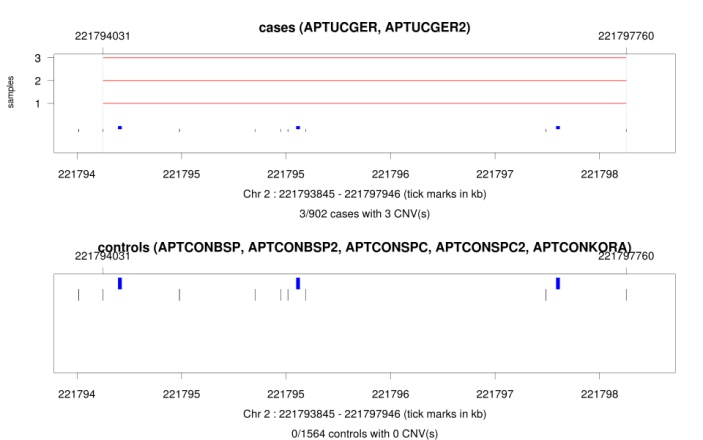

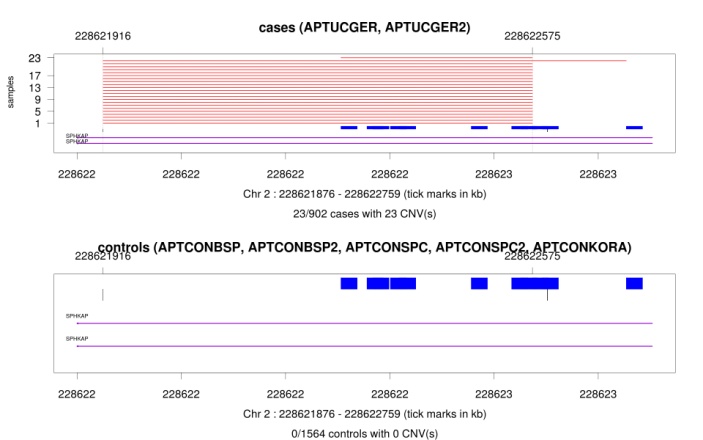

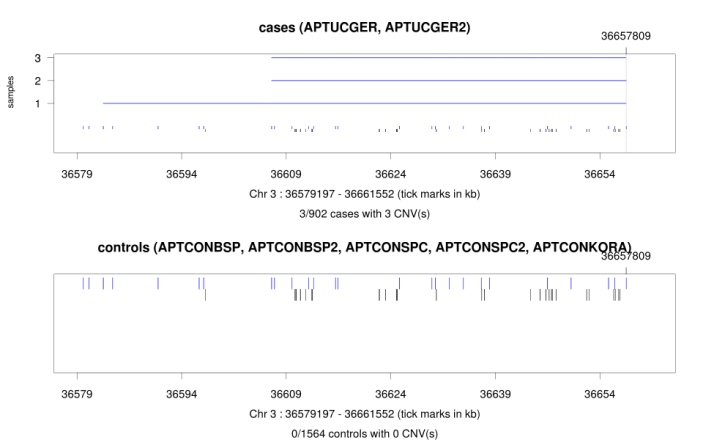

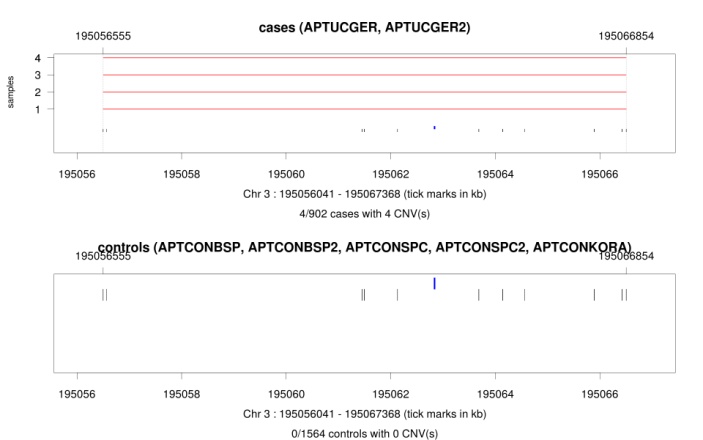

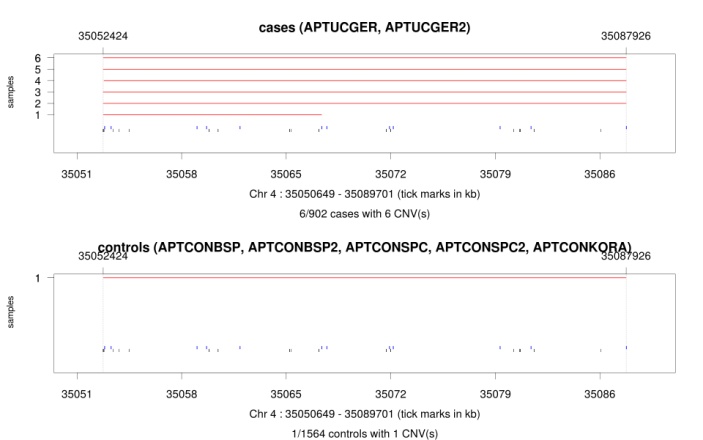

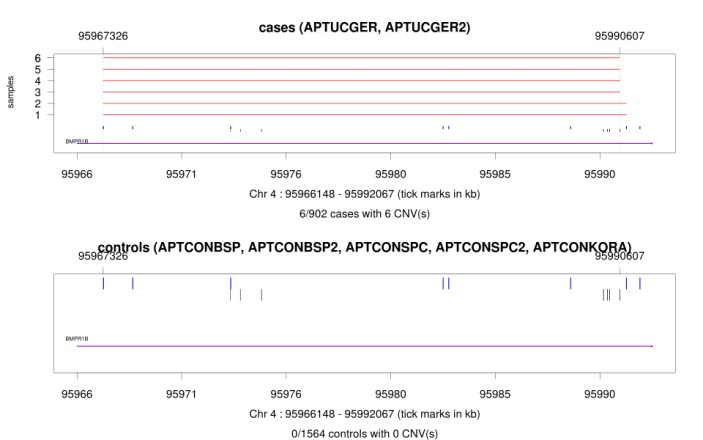

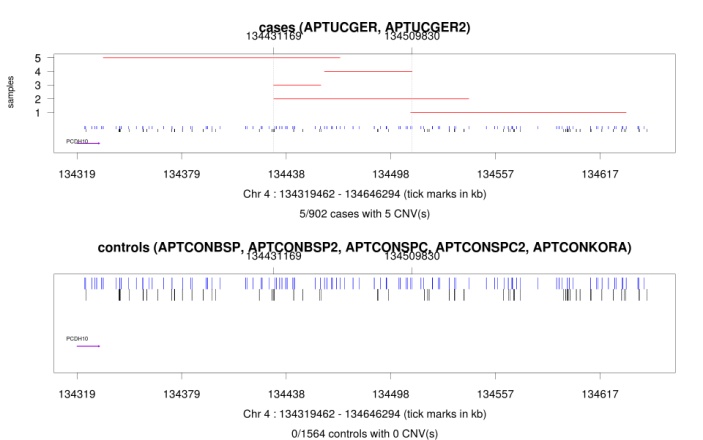

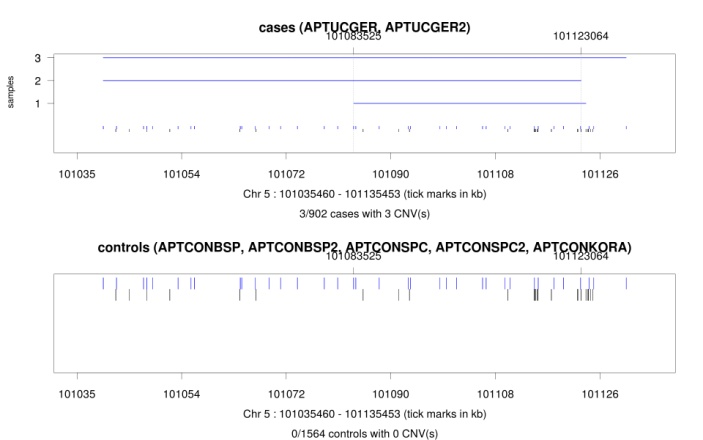

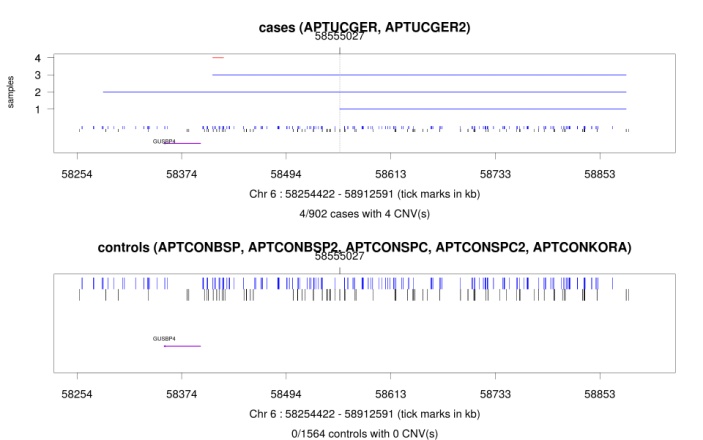

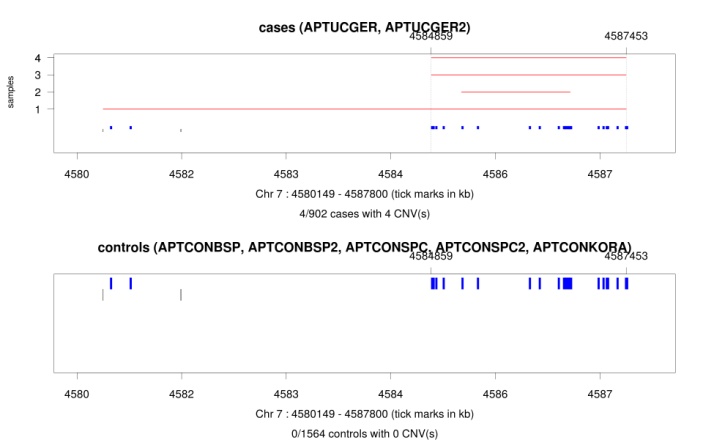

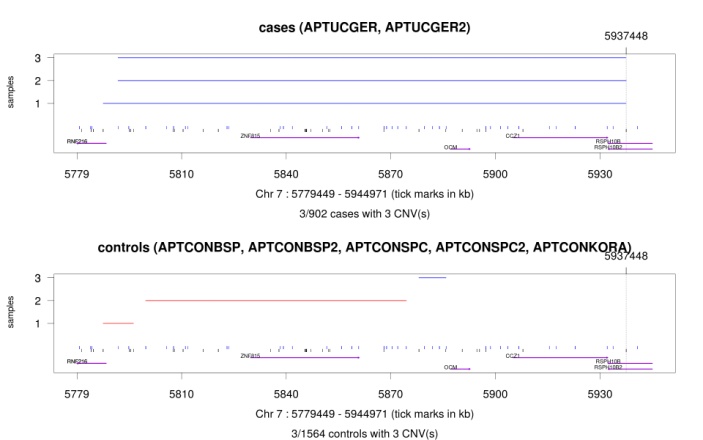

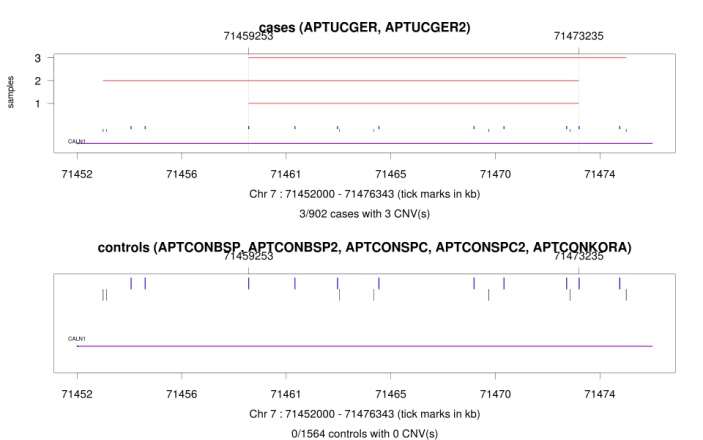

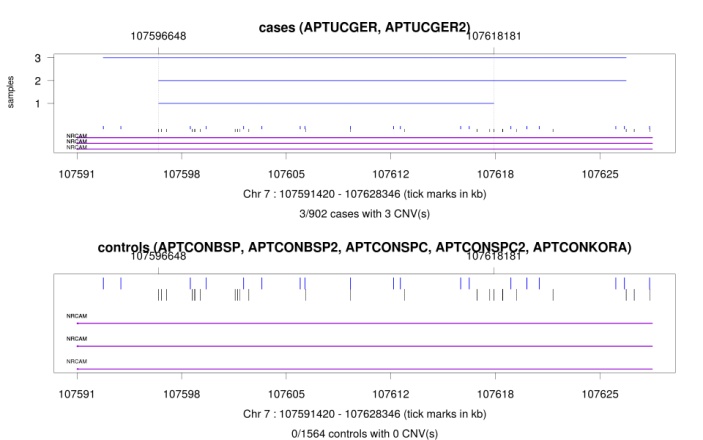

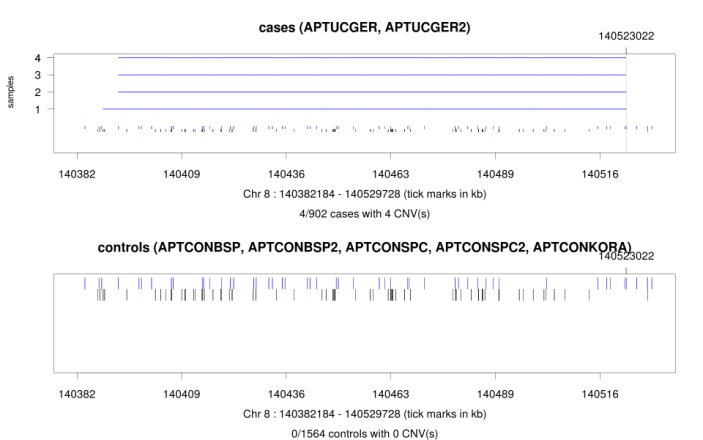

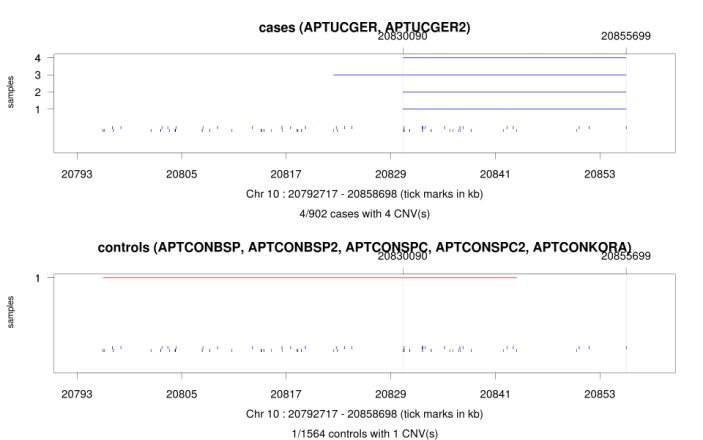

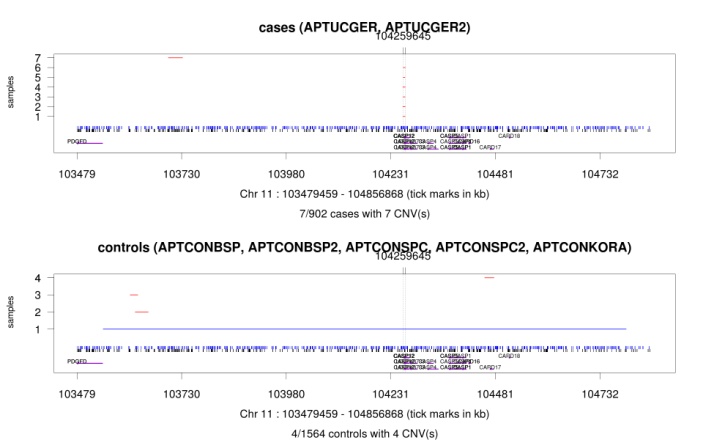

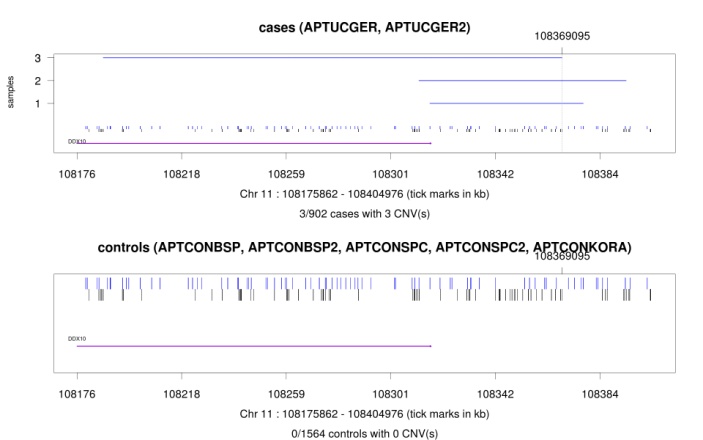

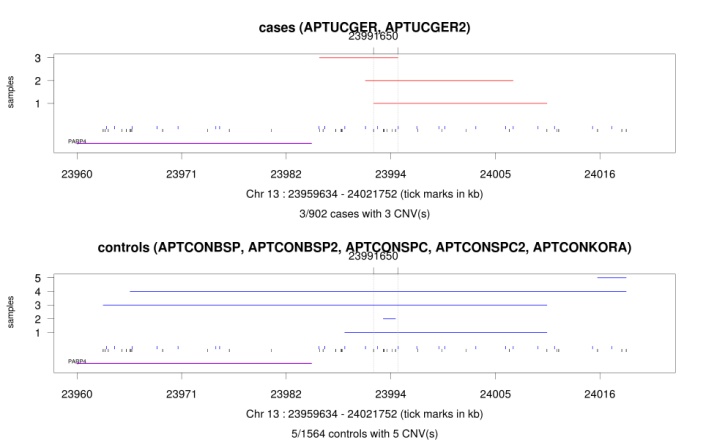

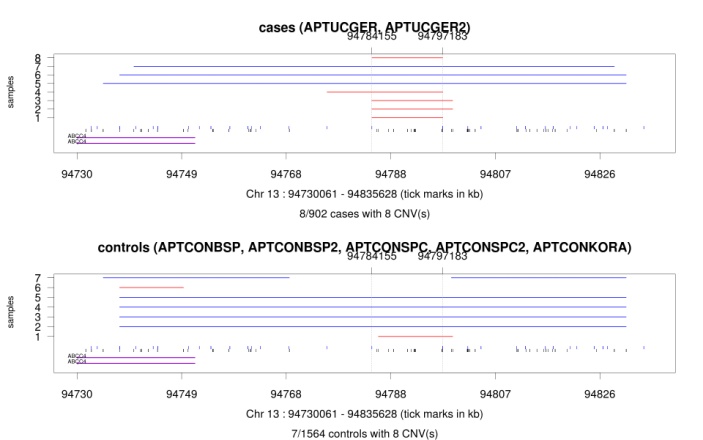

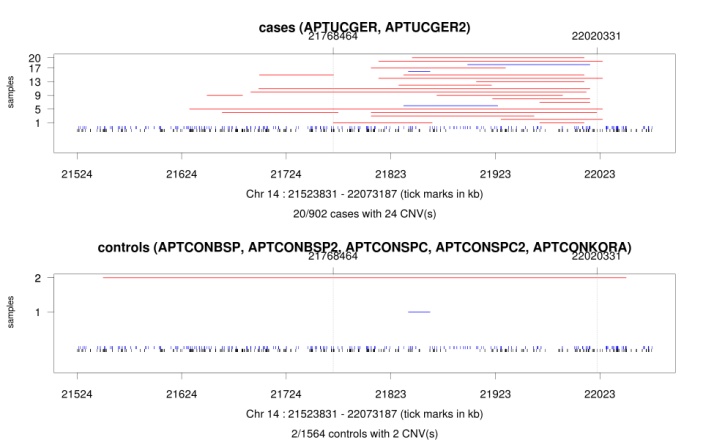

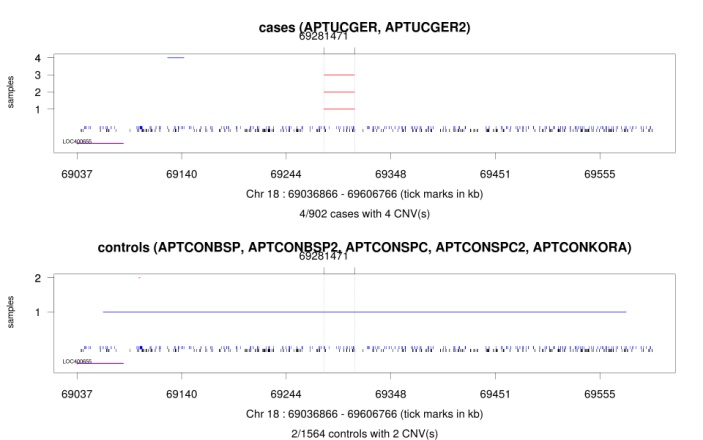

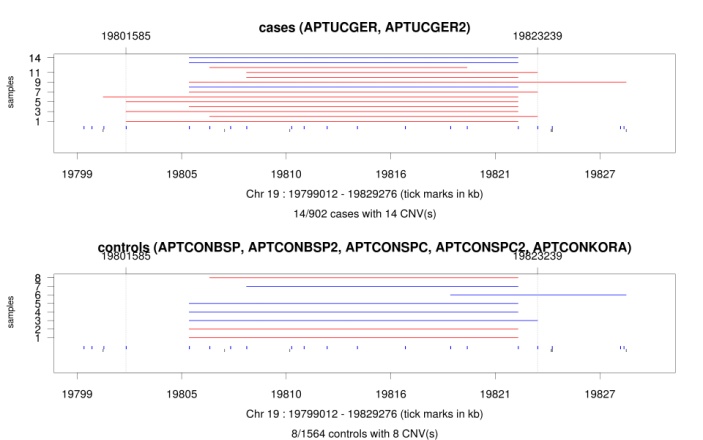

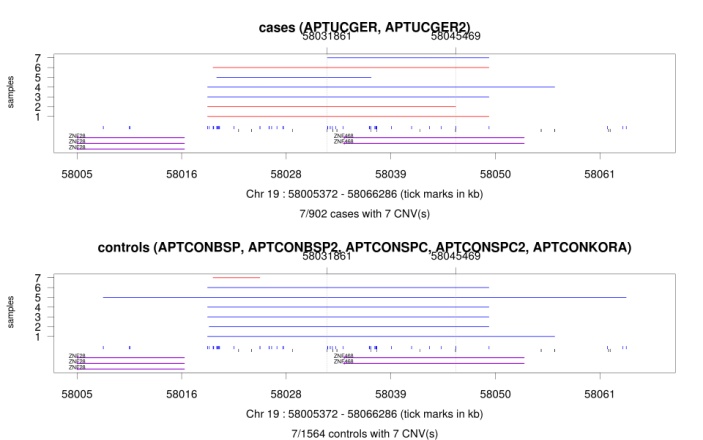


**Suppl. Table 1. Frequencies of the 24 identified CNVs in German discovery in comparison with the WTCCC2 panel.** For duplications the CNV counts are in blue, for deletions in red. The 2 duplications, overrepresented in cases (compared to controls) in both panels as well as the deletion (significant only in German discovery) are marked in green.

|  |  | screening sample | | WTCCC2 sample | | screening sample | | WTCCC2 sample | |
| --- | --- | --- | --- | --- | --- | --- | --- | --- | --- |
|  |  | CNV counts | | CNV counts | | CNV freq in % | | CNV freq in % | |
| genomic position | cytoband | 902 cases | 1564 controls | 2396 cases | 4886 controls | cases | controls | cases | controls |
| chr1:1151109-1151109 | 1p36.33 | **3** | **0** | **2** | **0** | 0.33 | 0.00 | 0.08 | 0.00 |
| chr2:221794031-221797760 | 2q36.1 | **3** | **0** | **7** | **11** | 0.33 | 0.00 | 0.29 | 0.23 |
| chr2:228621916-228622575 | 2q36.3 | **23** | **0** | **0** | **0** | 2.55 | 0.00 | 0.00 | 0.00 |
| chr3:195056555-195066854 | 3q29 | **4** | **0** | **0** | **1** | 0.44 | 0.00 | 0.00 | 0.02 |
| chr3:36657809-36657809 | 3p22.2 | **3** | **0** | **0** | **2** | 0.33 | 0.00 | 0.00 | 0.04 |
| chr4:134431169-134509830 | 4q28.3 | **4** | **0** | **1** | **4** | 0.44 | 0.00 | 0.04 | 0.08 |
| chr4:35052424-35087926 | 4p15.1 | **6** | **1** | **11** | **16** | 0.67 | 0.06 | 0.46 | 0.33 |
| chr4:95967326-95990607 | 4q22.3 | **6** | **0** | **0** | **1** | 0.67 | 0.00 | 0.00 | 0.02 |
| chr5:101083525-101123064 | 5q21.1 | **3** | **0** | **1** | **5** | 0.33 | 0.00 | 0.04 | 0.10 |
| chr6:58882675-58882675 | 6p11.1 | **3** | **0** | **2** | **5** | 0.33 | 0.00 | 0.08 | 0.10 |
| chr7:107596648-107618181 | 7q31.1 | **3** | **0** | **0** | **2** | 0.33 | 0.00 | 0.00 | 0.04 |
| chr7:4584859-4587453 | 7p22.1 | **4** | **0** | **0** | **0** | 0.44 | 0.00 | 0.00 | 0.00 |
| **chr7:5937448-5937448** | **7p22.1** | **3** | **0** | **3** | **0** | **0.33** | **0.00** | **0.13** | **0.00** |
| chr7:71459253-71473235 | 7q11.22 | **3** | **0** | **0** | **0** | 0.33 | 0.00 | 0.00 | 0.00 |
| **chr8:140523022-40523022** | **8q24.3** | **4** | **0** | **5** | **2** | **0.44** | **0.00** | **0.21** | **0.04** |
| chr10:20830090-20855699 | 10p12.31 | **4** | **0** | **8** | **18** | 0.44 | 0.00 | 0.33 | 0.37 |
| chr11:104259645-104265284 | 11q22.3 | **6** | **0** | **5** | **4** | 0.67 | 0.00 | 0.21 | 0.08 |
| chr11:108369095-108369095 | 11q22.3 | **3** | **0** | **0** | **0** | 0.33 | 0.00 | 0.00 | 0.00 |
| chr13:23991650-23994304 | 13q12.12 | **3** | **0** | **2** | **1** | 0.33 | 0.00 | 0.08 | 0.02 |
| **chr13:94784155-94797183** | **13q32.1** | **5** | **1** | **5** | **6** | **0.55** | **0.06** | **0.21** | **0.12** |
| chr14:21768464-22020331 | 14q11.2 | **17** | **1** | **42** | **28** | 1.88 | 0.06 | 1.75 | 0.57 |
| chr18:69281471-69311825 | 18q22.3 | **3** | **0** | **0** | **7** | 0.33 | 0.00 | 0.00 | 0.14 |
| chr19:19801585-19823239 | 19p12 | **11** | **3** | **6** | **4** | 1.22 | 0.19 | 0.25 | 0.08 |
| chr19:58031861-58045469 | 19q13.41 | **3** | **0** | **2** | **10** | 0.33 | 0.00 | 0.08 | 0.20 |

**Suppl. Figure 2. Independent Replication of Deletion 13q32.1 with TaqMan^®^** **CNV assays.** Each graph of the figure shows the result of real time PCR for one TaqMan^®^ plate. Graphs with red frame are for case plates and blue frames from control plates. Deletion carriers are highlighted by red bars. Samples with green bars are calibrator samples, which are samples with confirmed deletion. These calibrator samples are used to identify the CNV carriers within the replication sample set. If there is a second bar next to a red bar with the same identifier, then it is just a technical replicate. This was sometimes done due to the noisy nature of the data. The two technical replicates of the fourth graph (id 4020) show both copy number state one. Nevertheless this sample was not counted as deletion carrier, because it shows copy number state one for every TaqMan^®^ assay that ran on this plate. TaqMan^®^ quality control was based on confidence greater or equal to 94%.


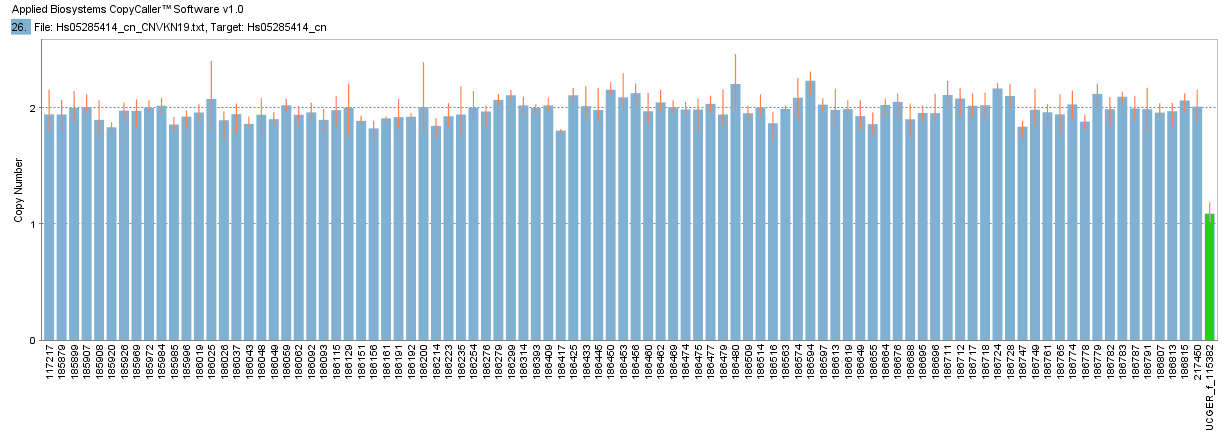

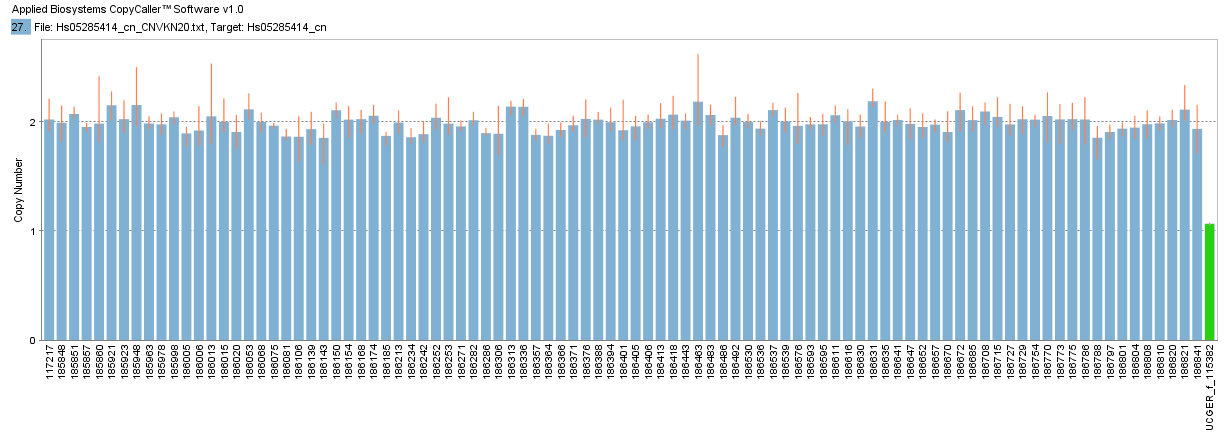


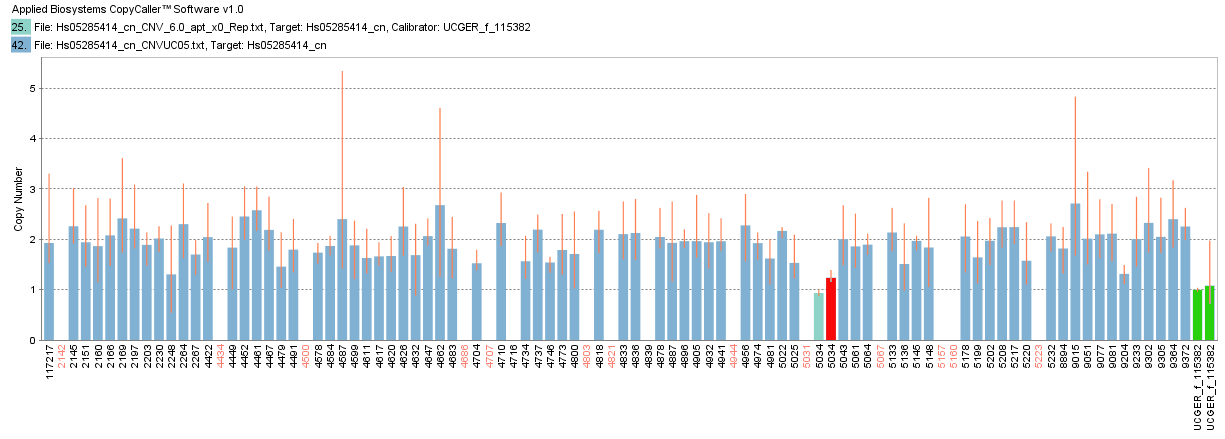

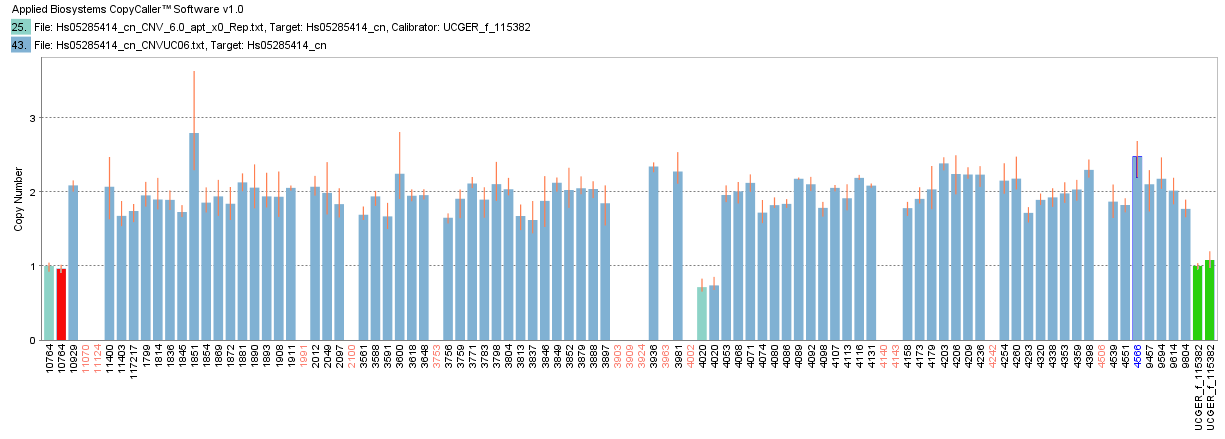


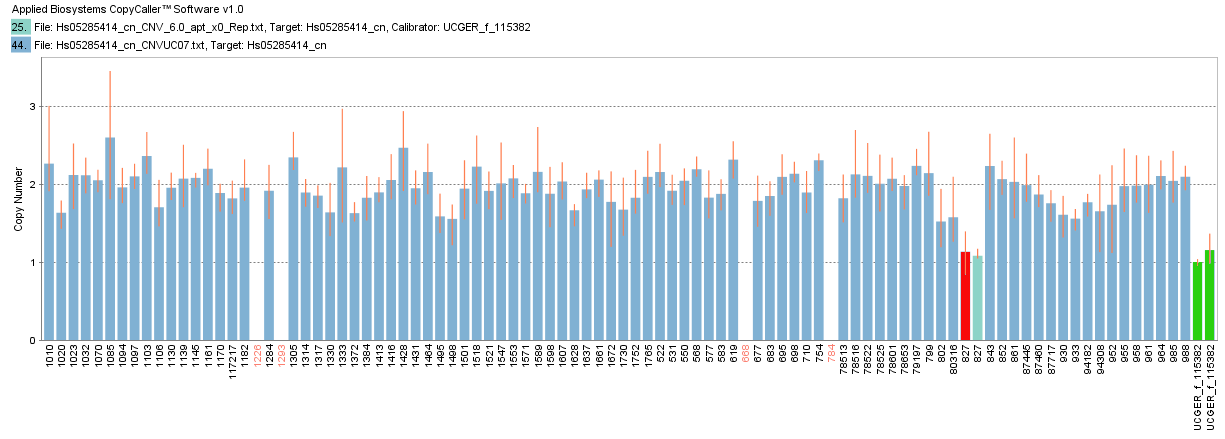

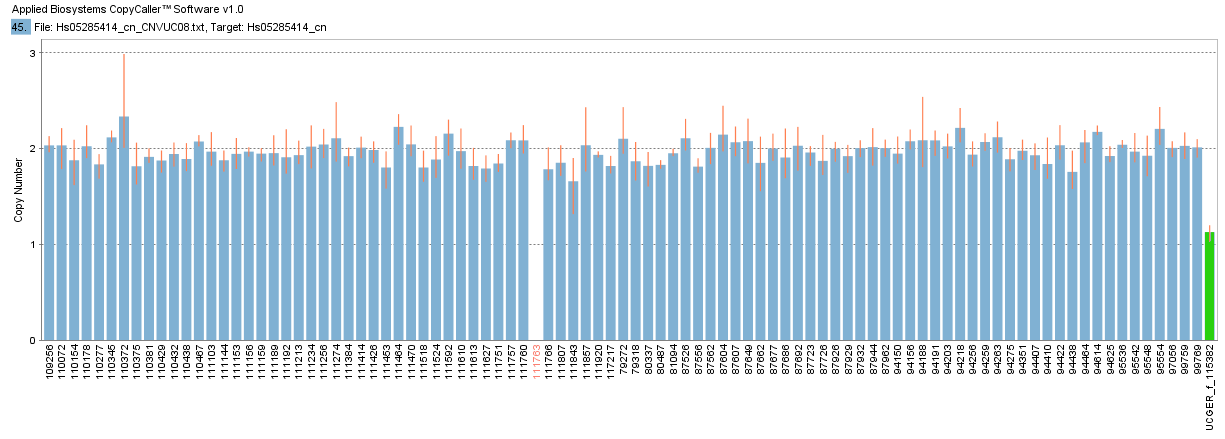


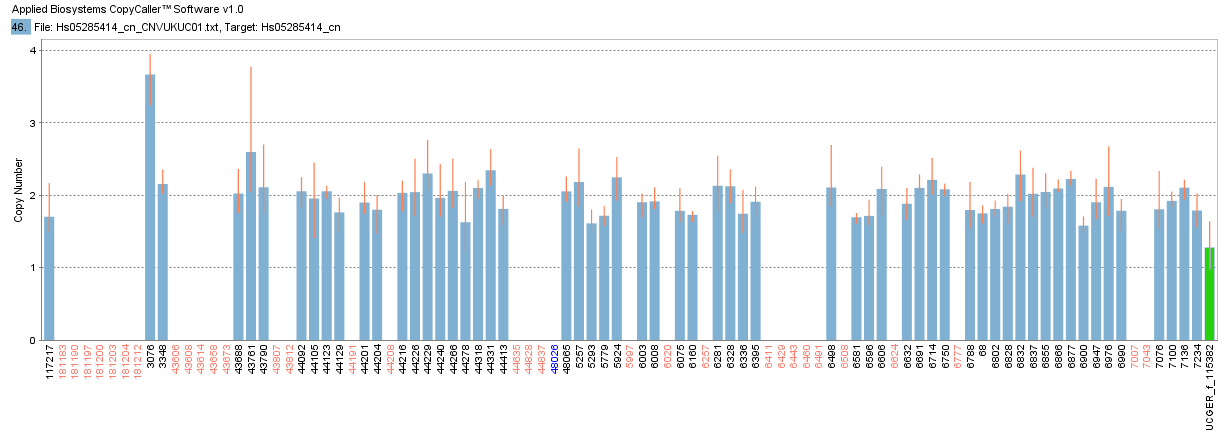

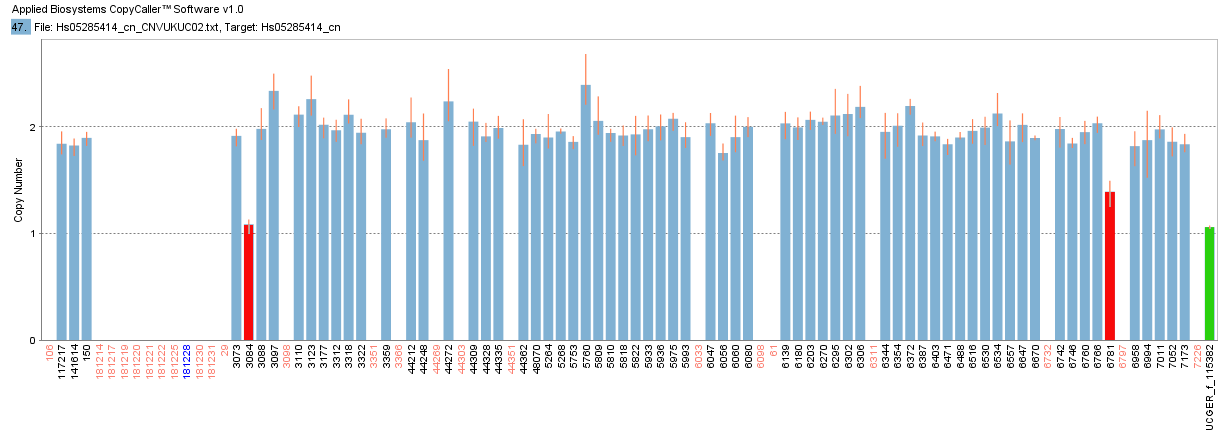


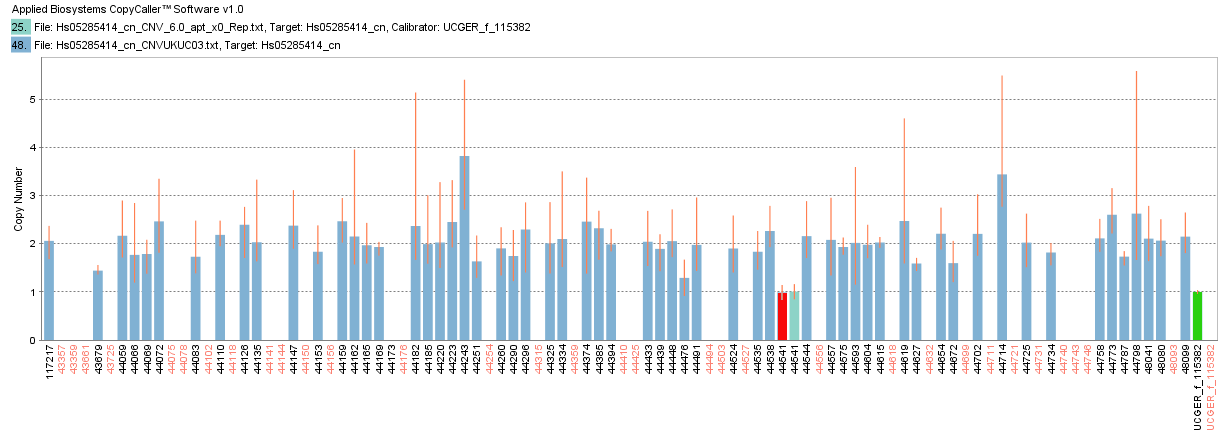


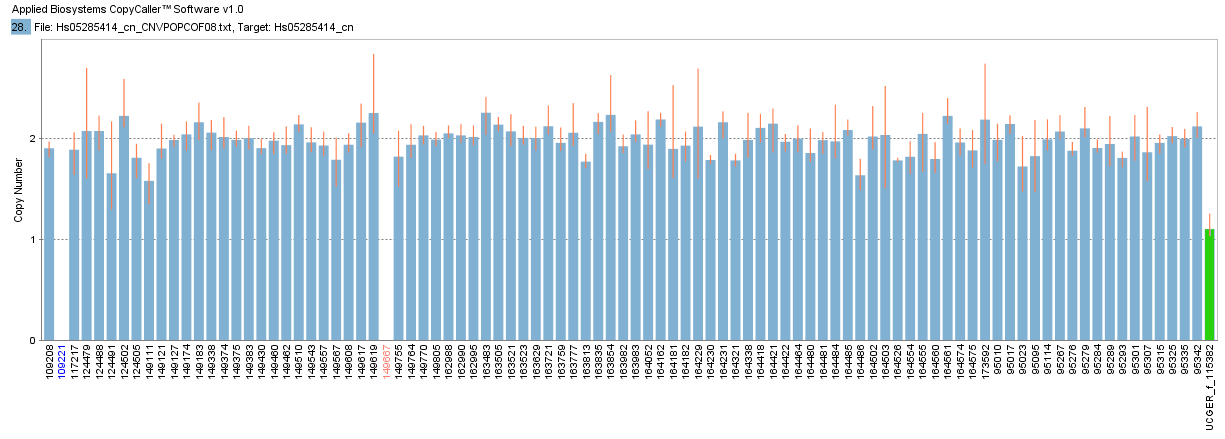

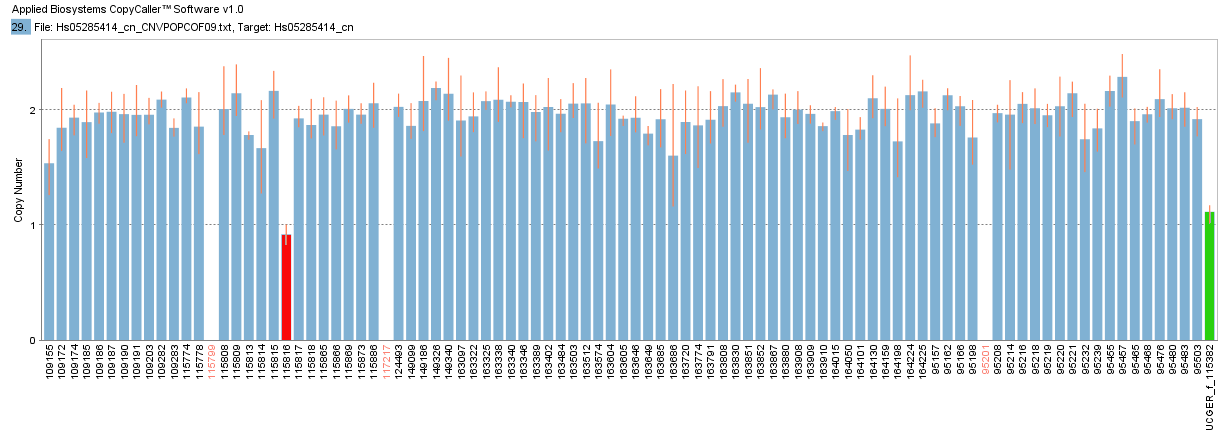


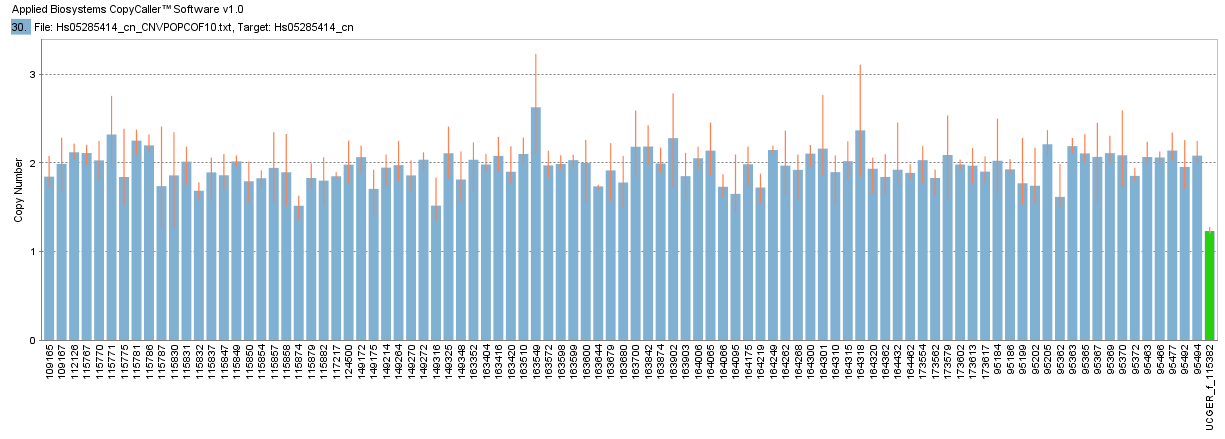

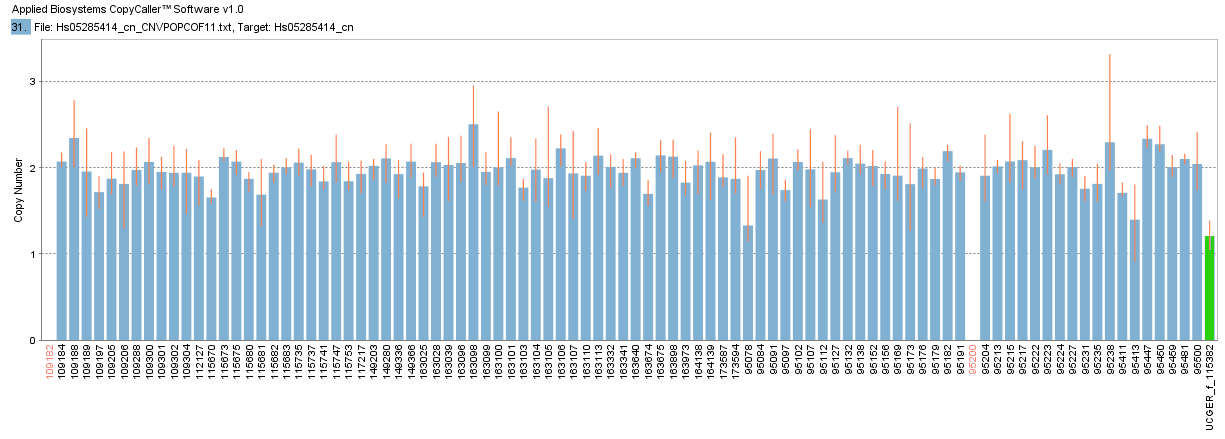


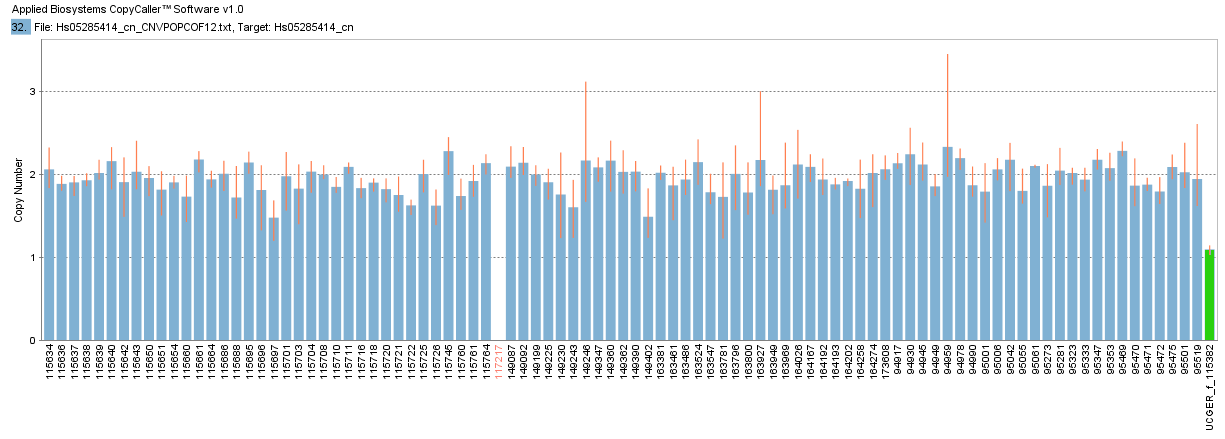

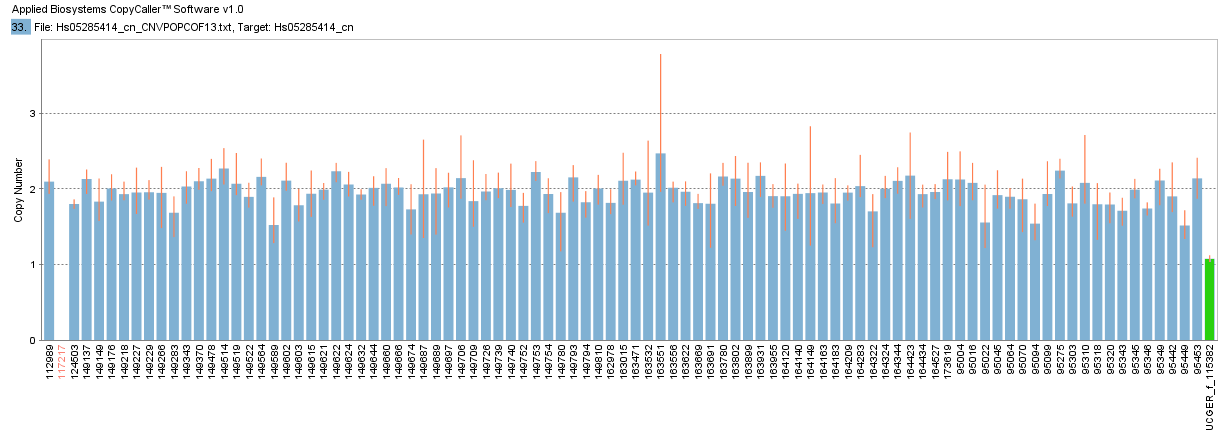


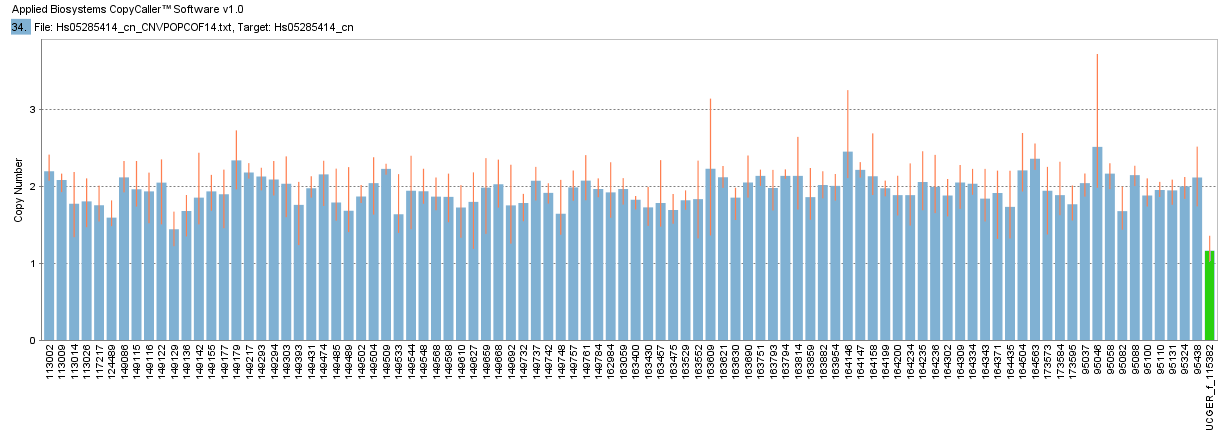

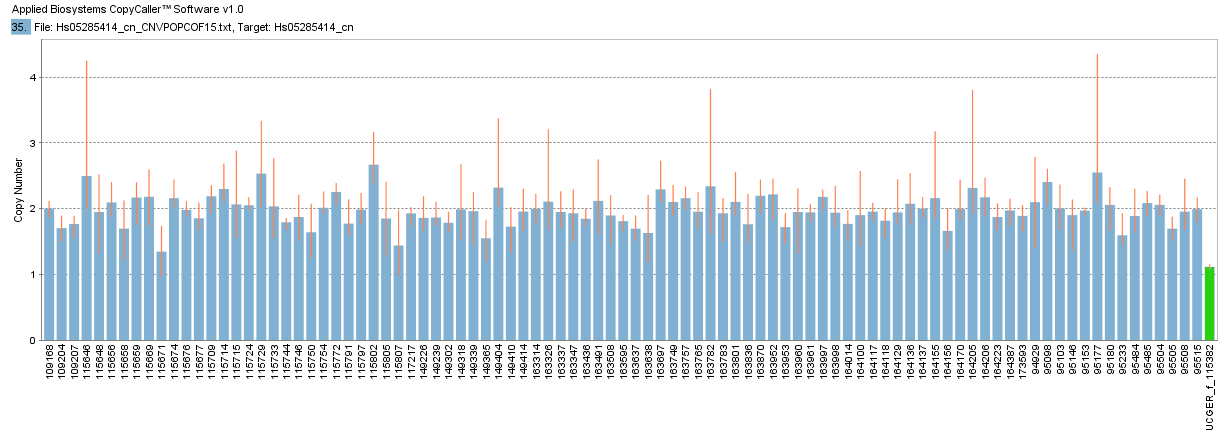


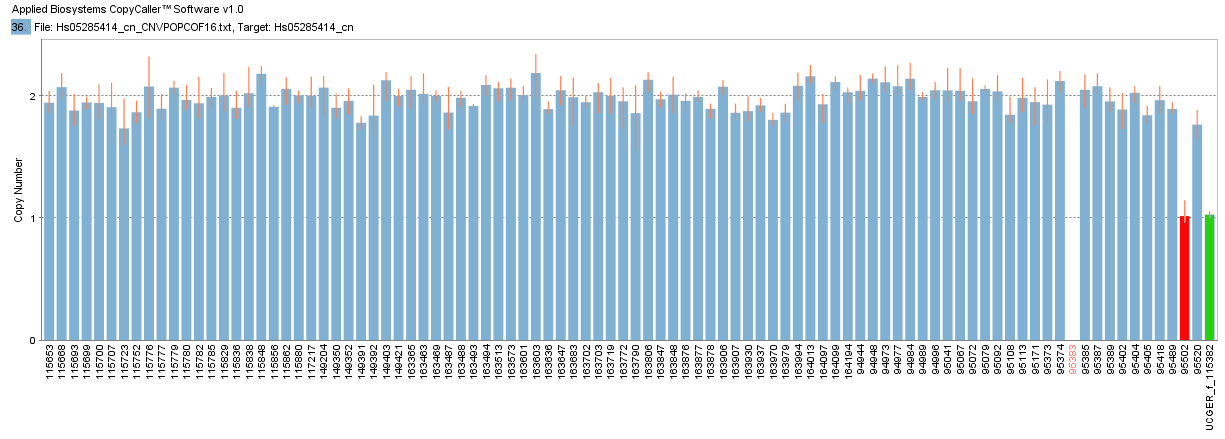

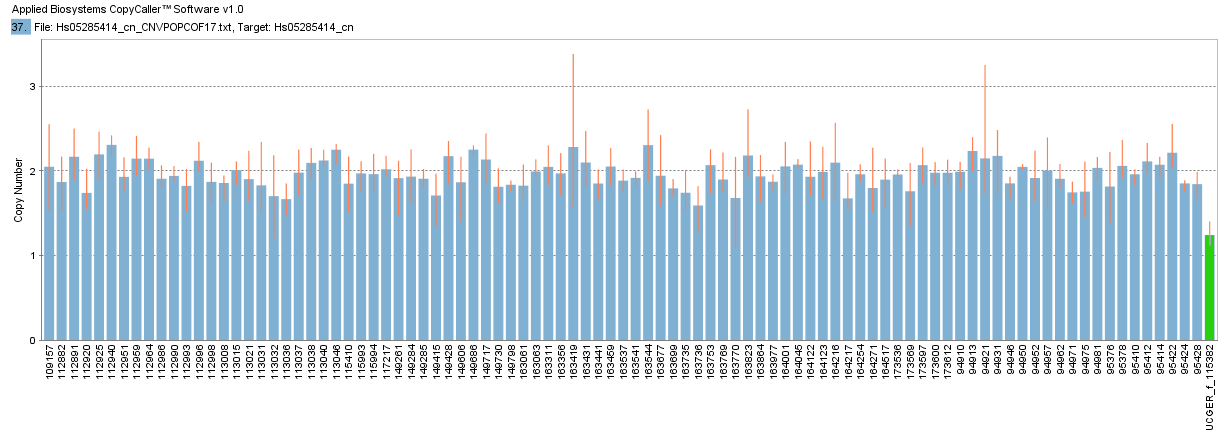


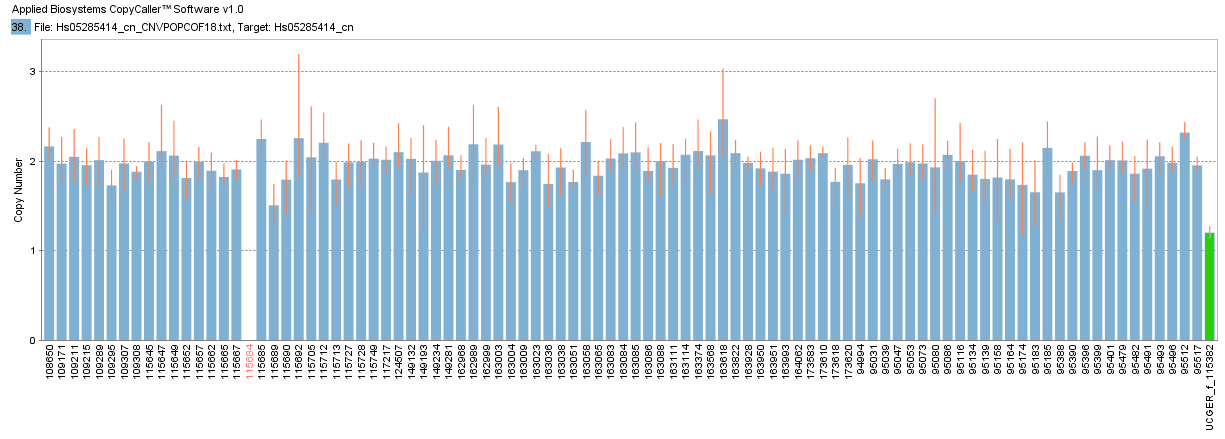

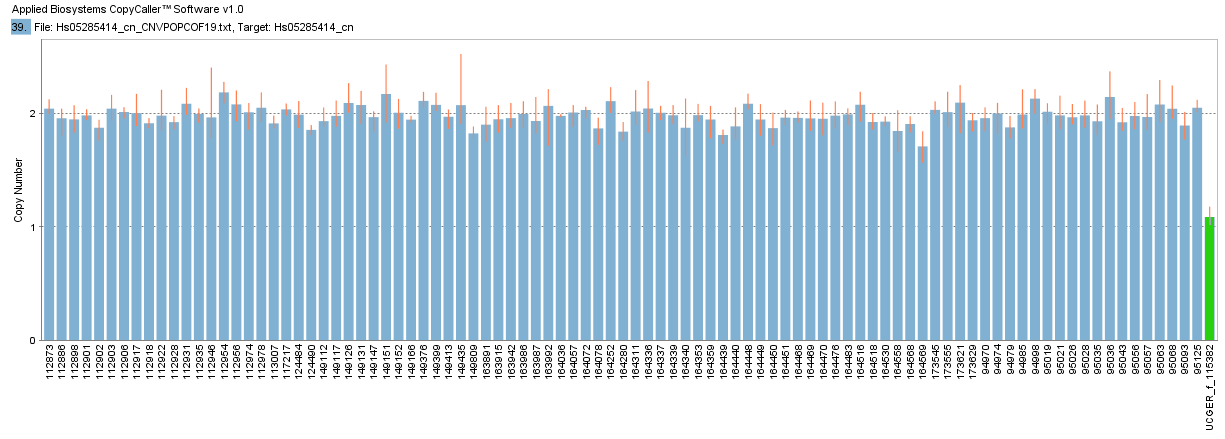


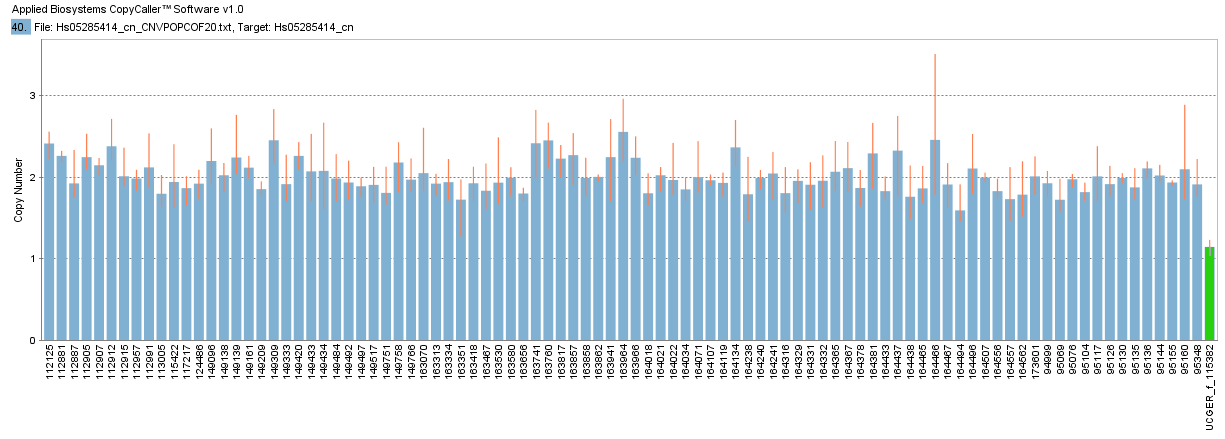

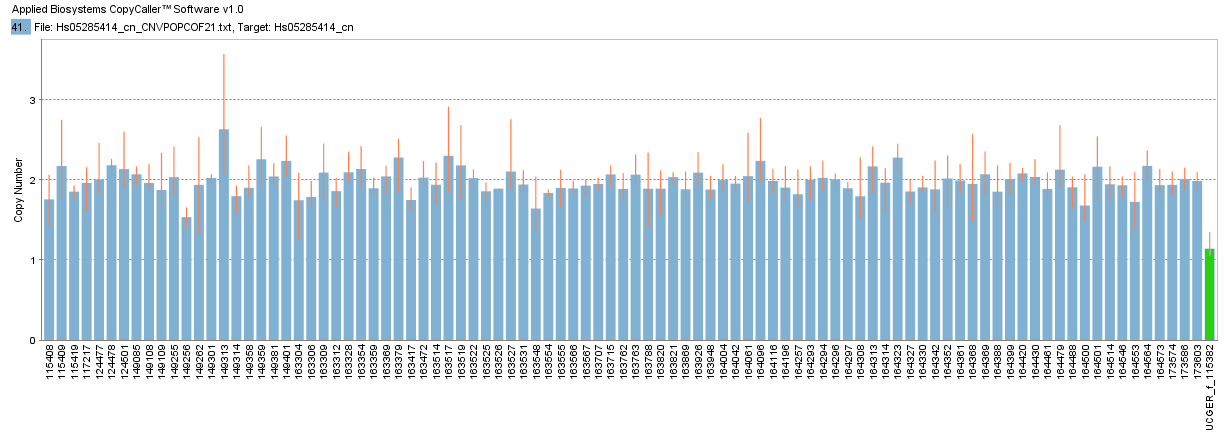


**Suppl. Figure 3. Technical validations of the three relevant CNVs (one deletion and 2 duplications) for the German discovery panel**

The top left picture of each of A-C shows the results of TaqMan^®^ CNV assay. While the majority of samples have copy number state two (blue bars). Green bars show deletion or duplication carriers. The top right picture of each A-C illustration shows the Affymetrix Power Tools (APT) prediction of CNVs for each region.The remaining pictures within each panel (A-C) show the raw data visualization of intensities as LRR in the top part and B allele frequency (BAF) in the lower part. Non polymorphic probe sets are blue and SNP probesets black, a smoothed spline was added for all LRR plots. RefGene annotation is added with purple arrows. The red (deletion) or blue (duplication) horizontal bars visualize the predicted CNV.

**(A)** **Del13q32.1**

The TaqMan^®^ results show exact correlation with the predicted CNVs and raw data quality of the SNP array data is pretty good. For all deletions a loss of signal intensity can be seen corresponding with a loss of heterozygosity.


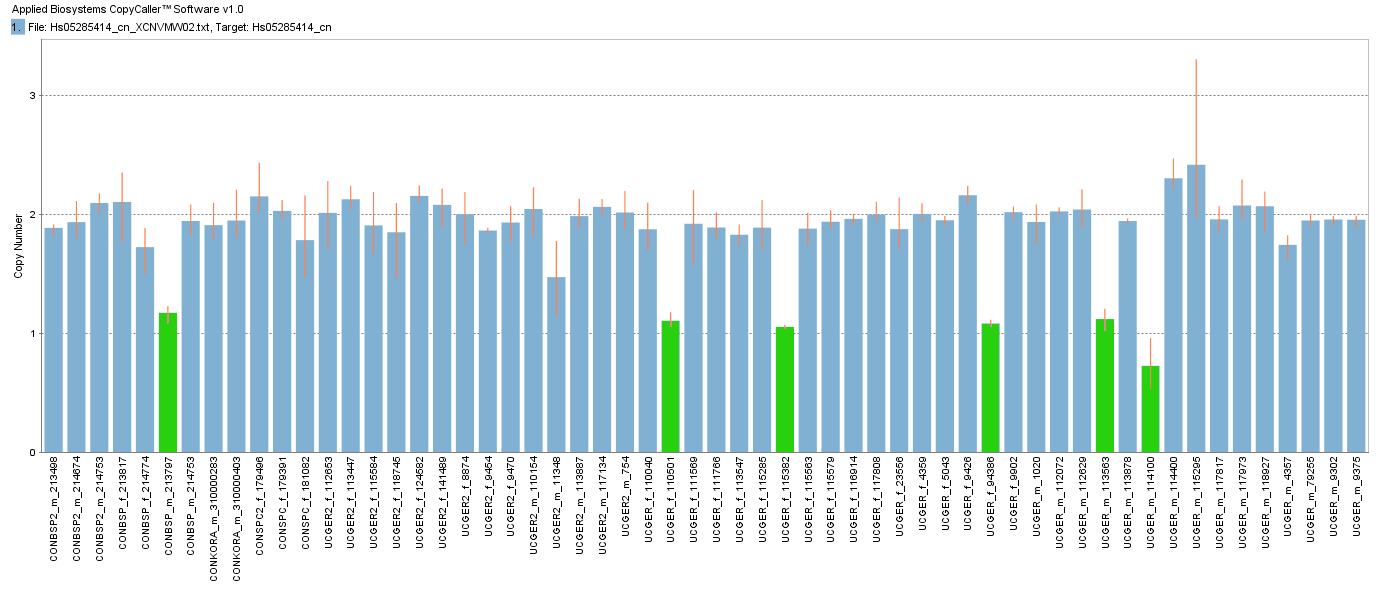

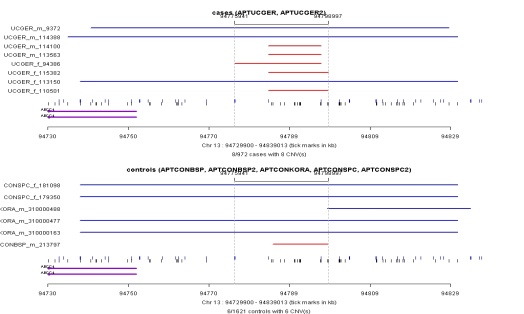


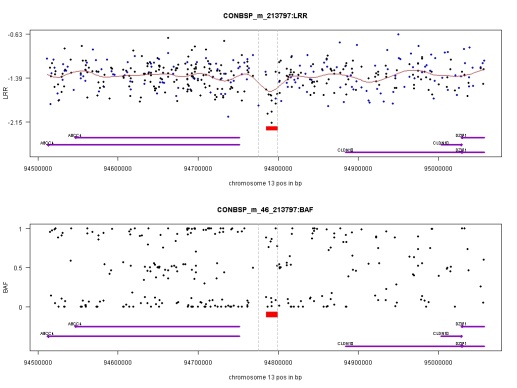

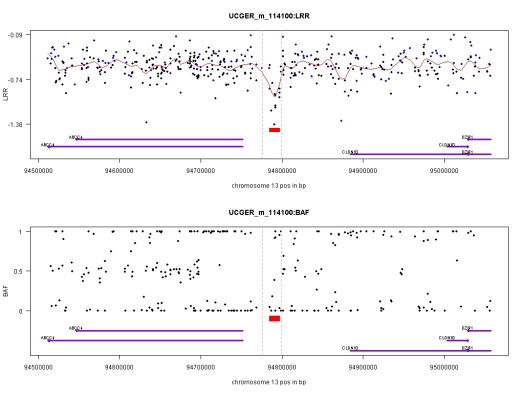

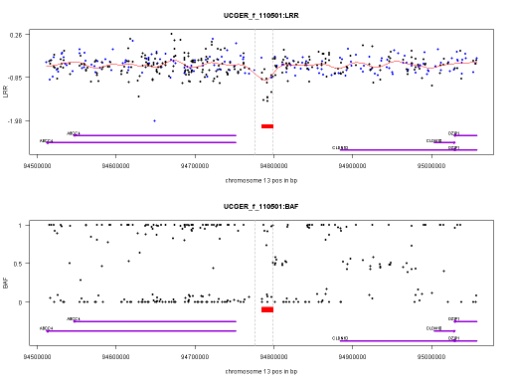


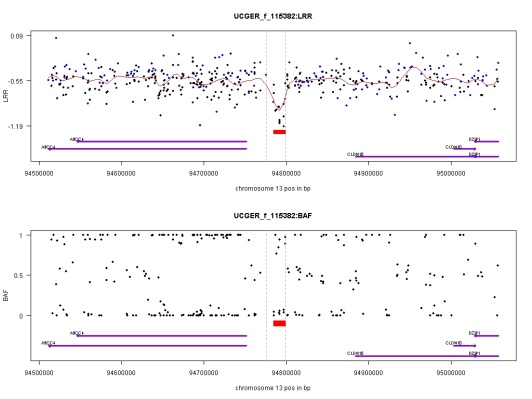

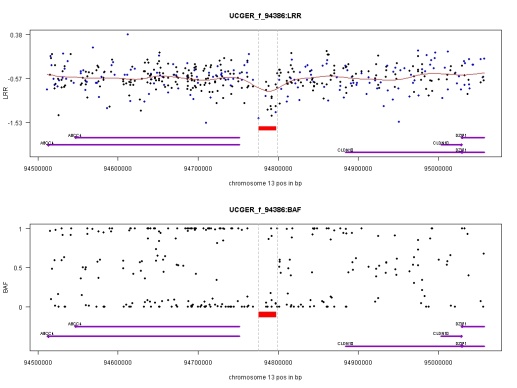

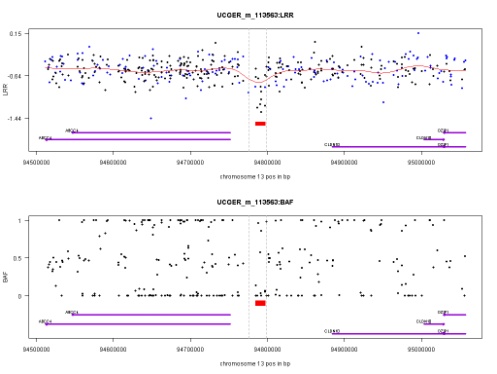


**(B) Dup7p22.1** The TaqMan^®^ results show exact correlation with the predicted CNVs and raw data quality of the SNP array data is pretty good. For all duplication clearly stronger signal intensity can be seen corresponding with a change of BAF. Instead of two homozygotes and one heterozygote part, within the predicted duplication a more complex signal distribution could be seen. This would be expected as for the duplicated are the genotypes AAA, AAB, ABB and BBB are possible for each SNP.


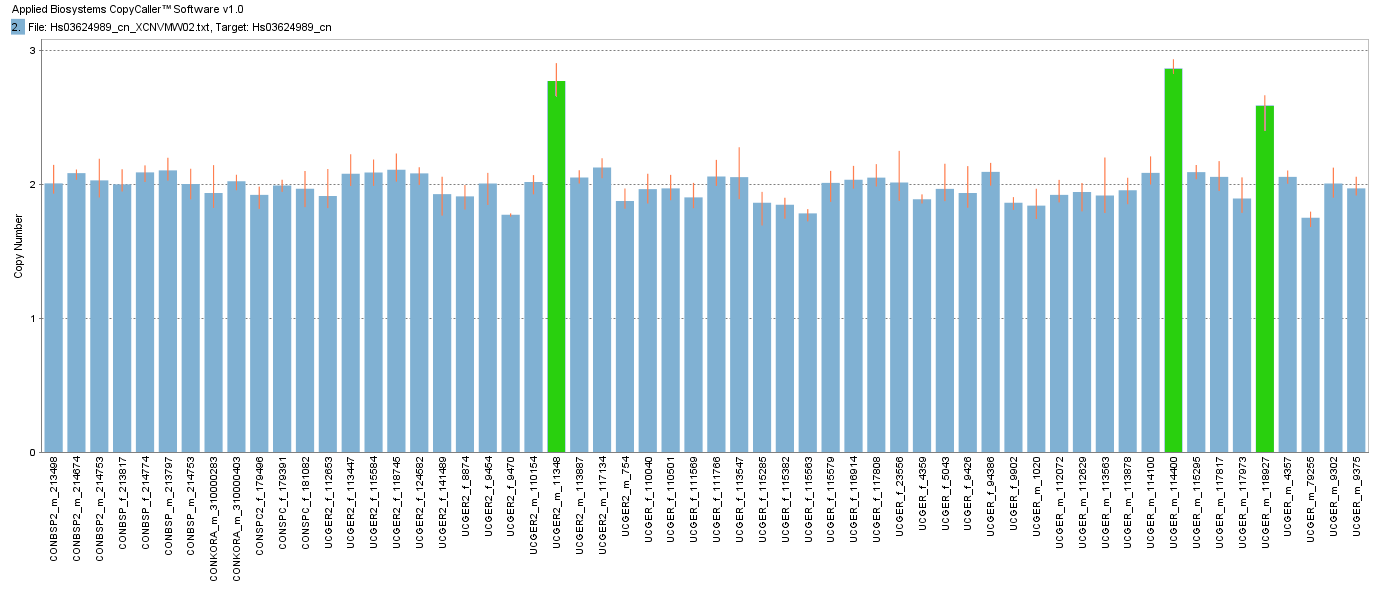

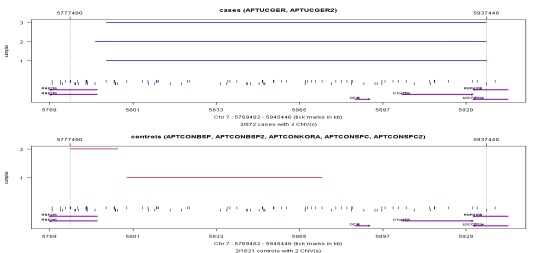


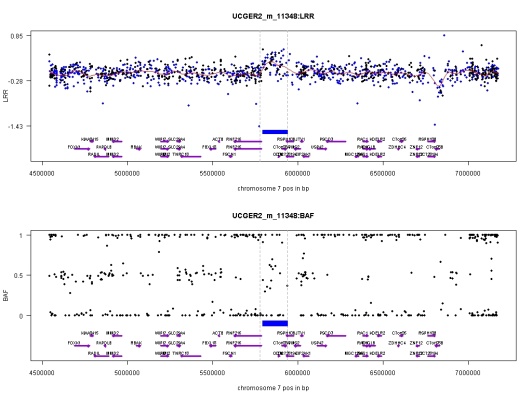

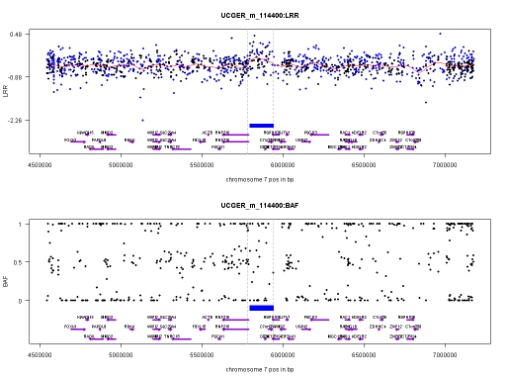

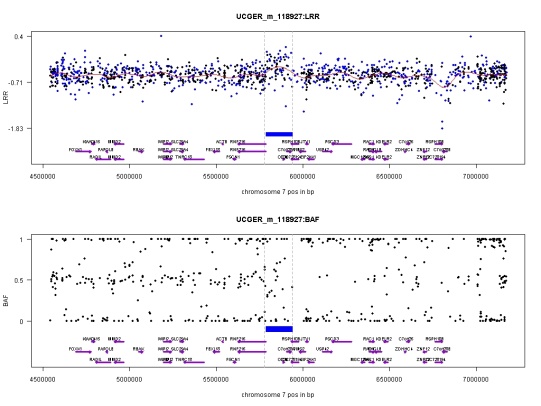


**(C)** **Dup8q24.3**


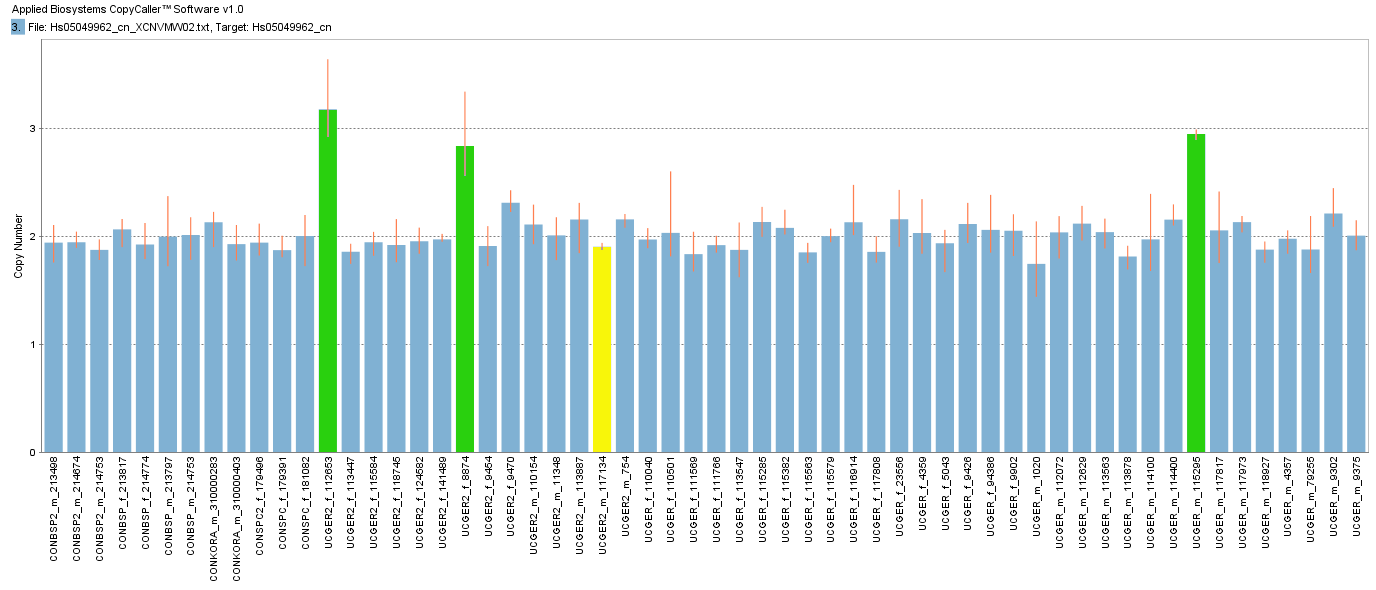

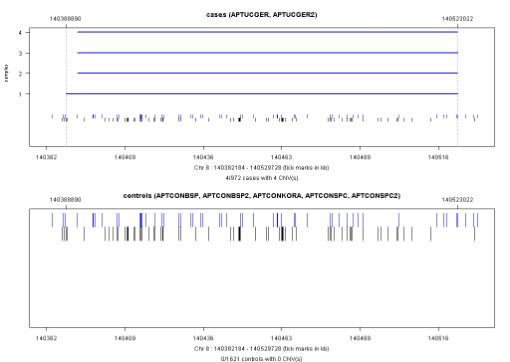


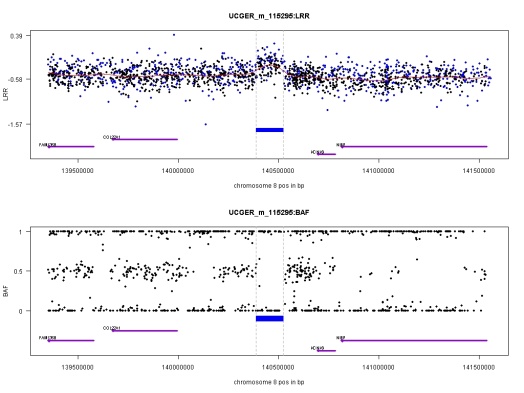

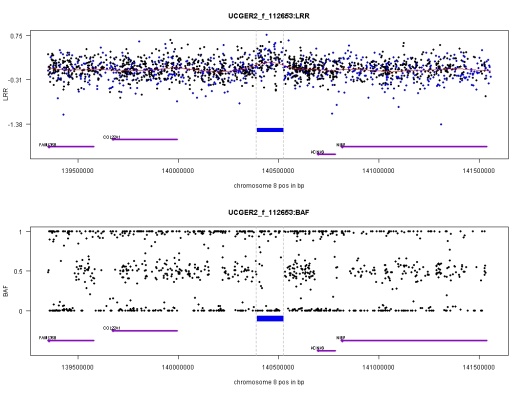

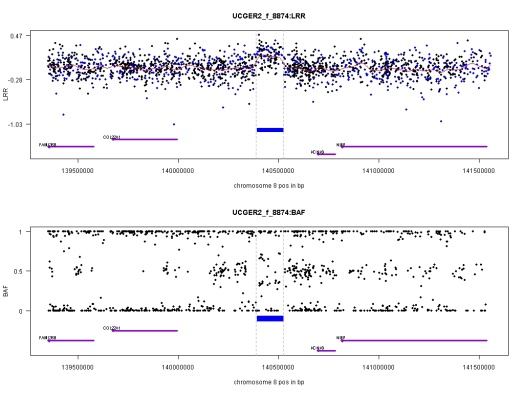


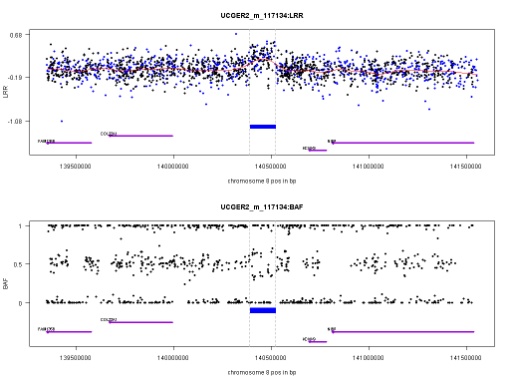


**Suppl. Figure 4. Independent Replication of Duplication 7p22.1 with TaqMan^®^ CNV assays.** Each graph of the figure shows the result of one real time PCR TaqMan^®^ plate, with the samples at the x-axis and the intensities at the y-axis. Graphs with red frame are from case plates, blue frames from control plates and green frames from technical replicates. Duplication carriers are highlighted by dark blue bars. Samples with green bars are calibrator samples, which are samples with confirmed duplication. These calibrator samples are used to identify the CNV carrier within the replication sample set. If there is a second bar next to a dark blue bar with the same identifier, then it is just a technical replicate. This was sometimes done due to the noisy nature of the data. TaqMan^®^ quality control was based on confidence greater or equal to 94%.


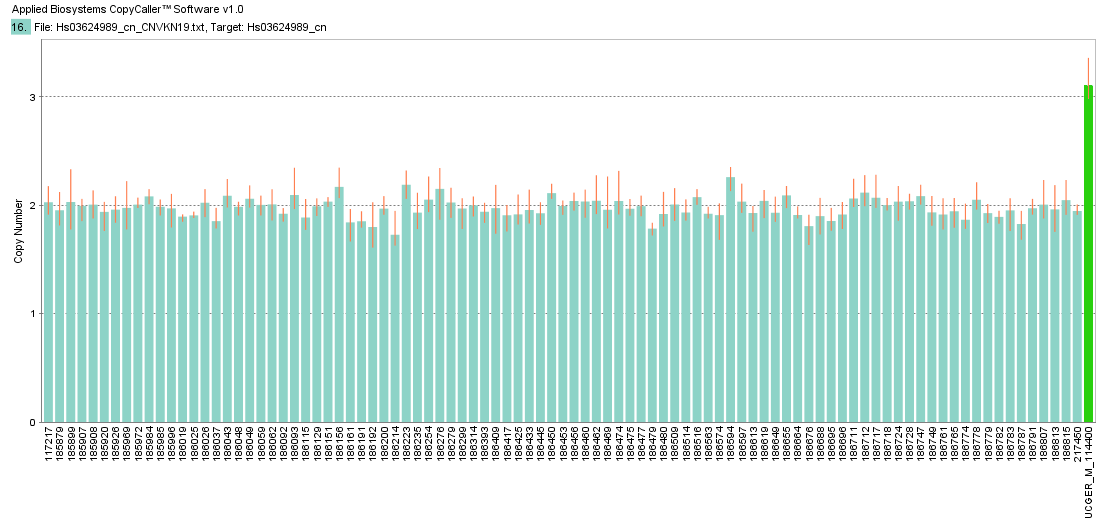

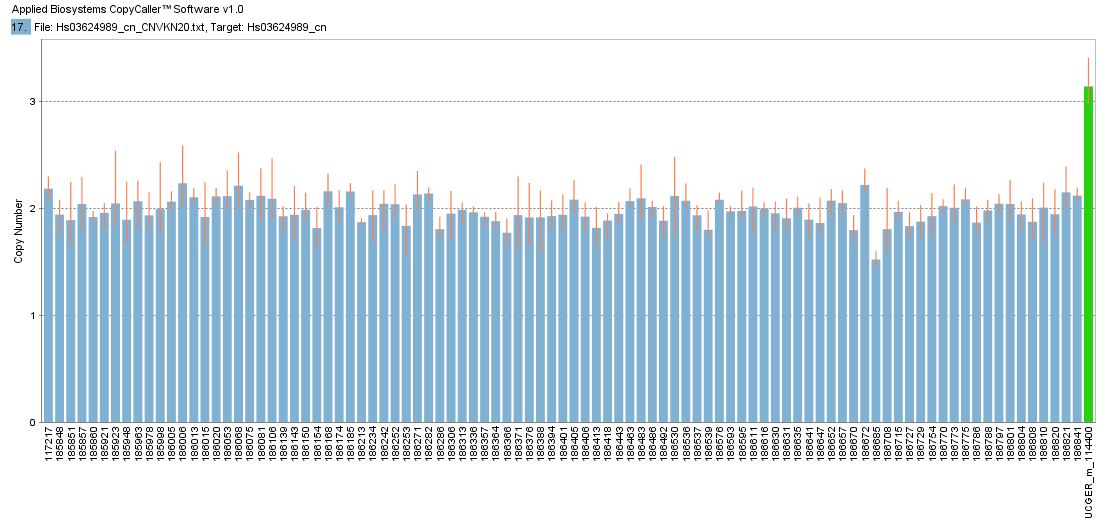


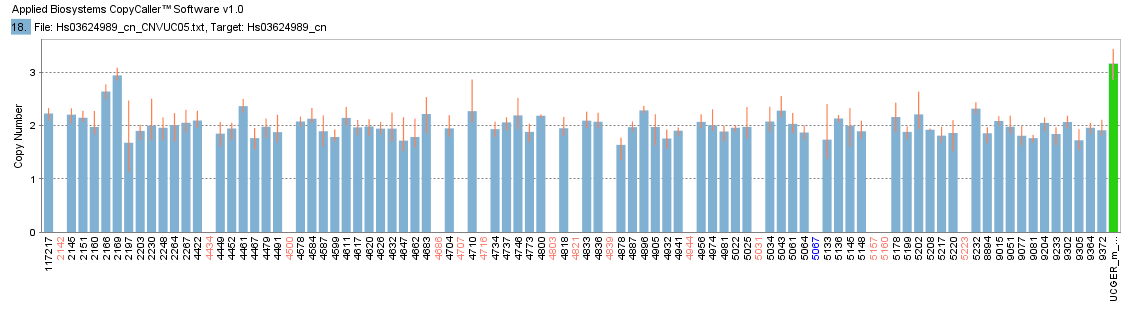

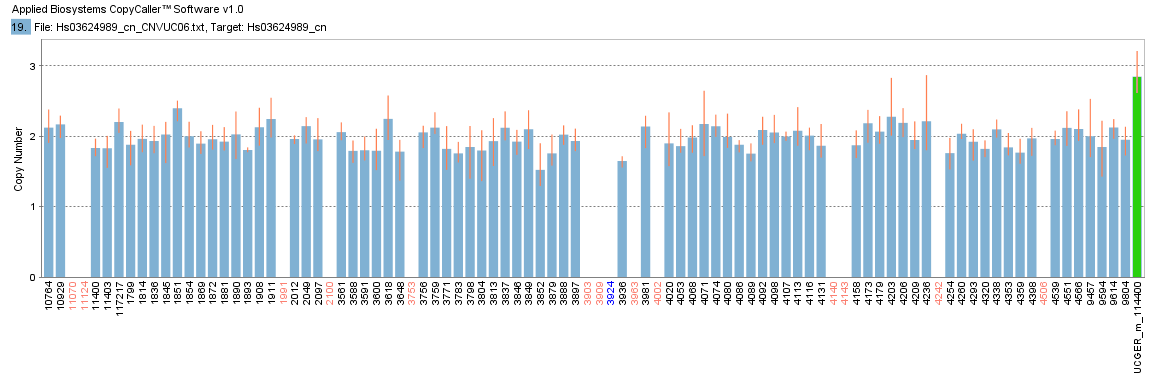


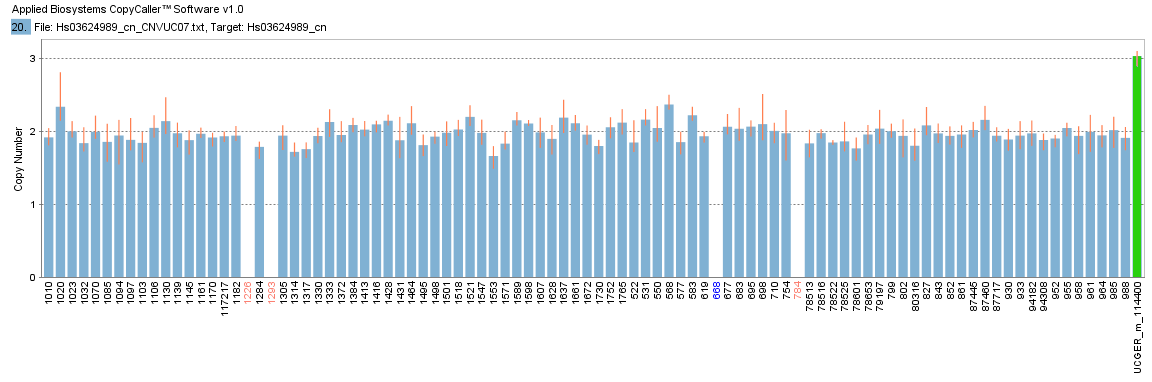

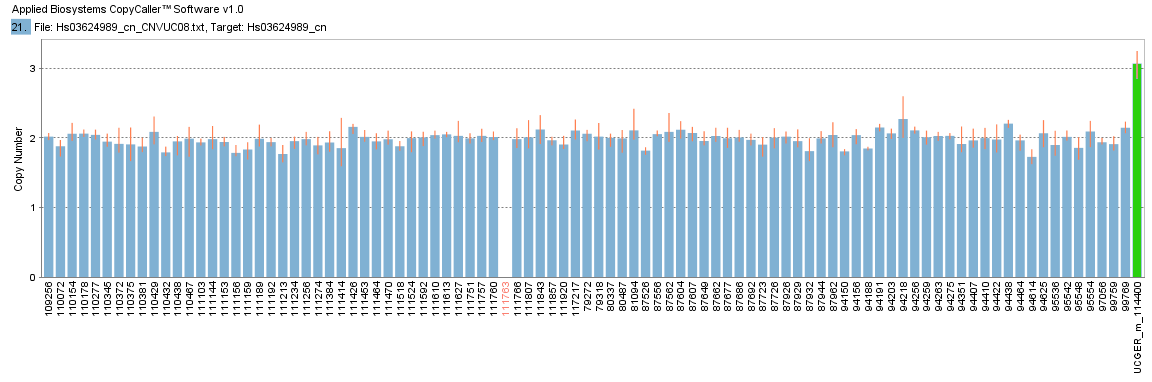


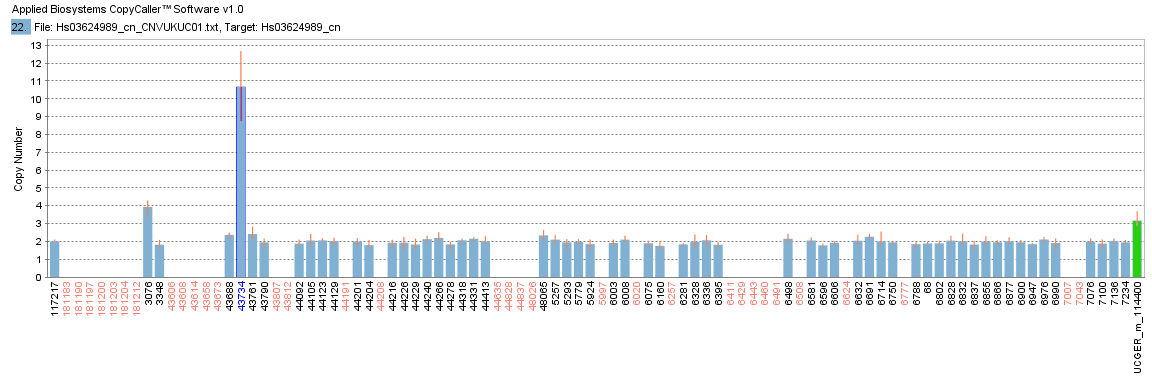

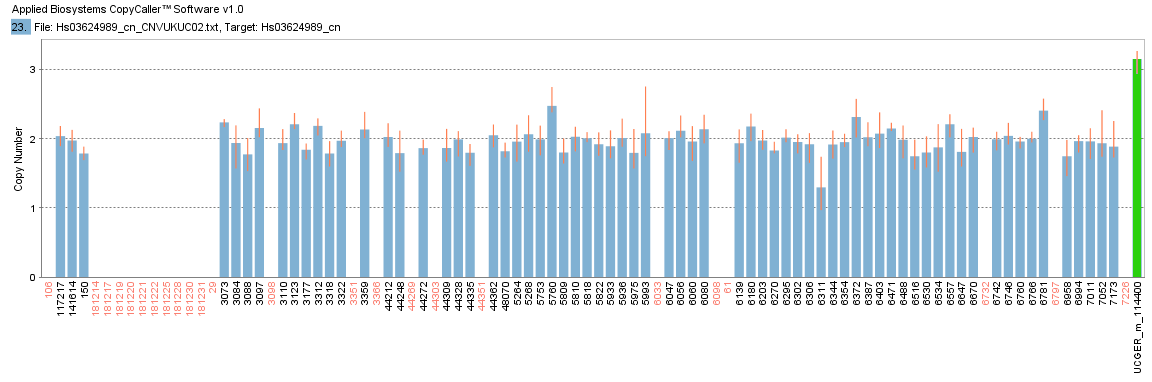


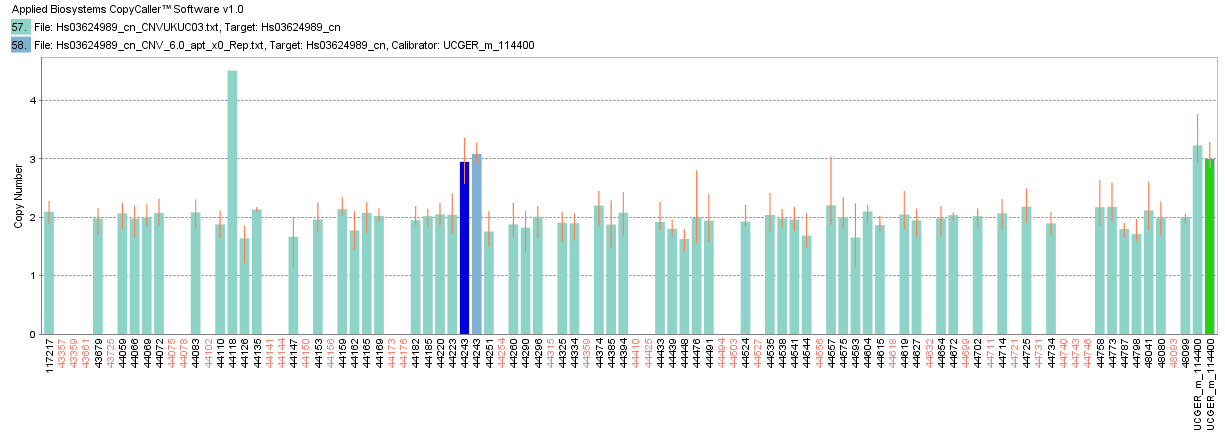


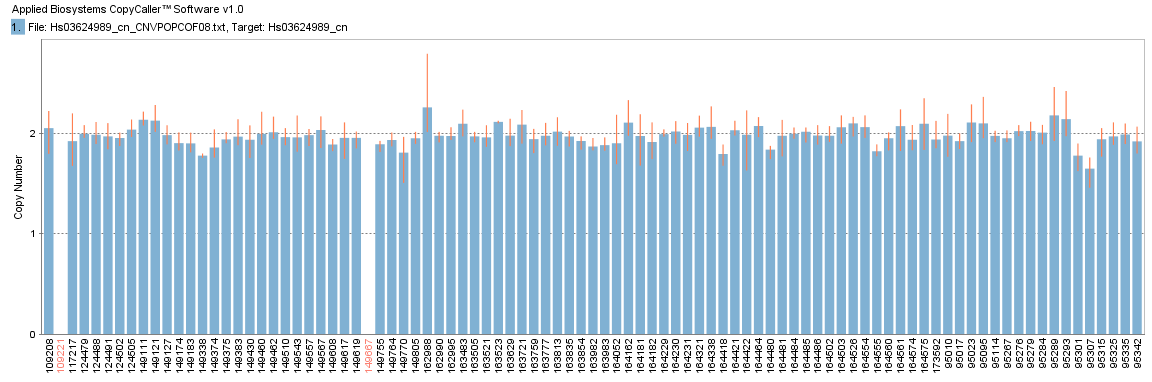

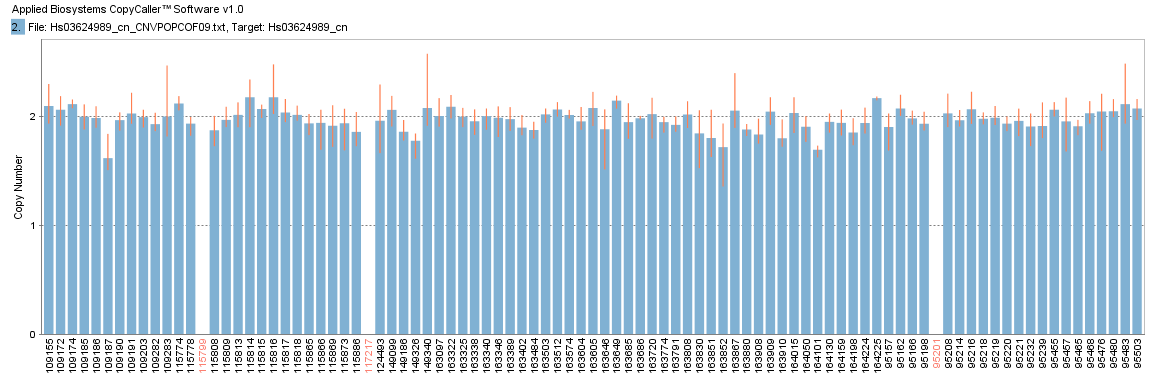


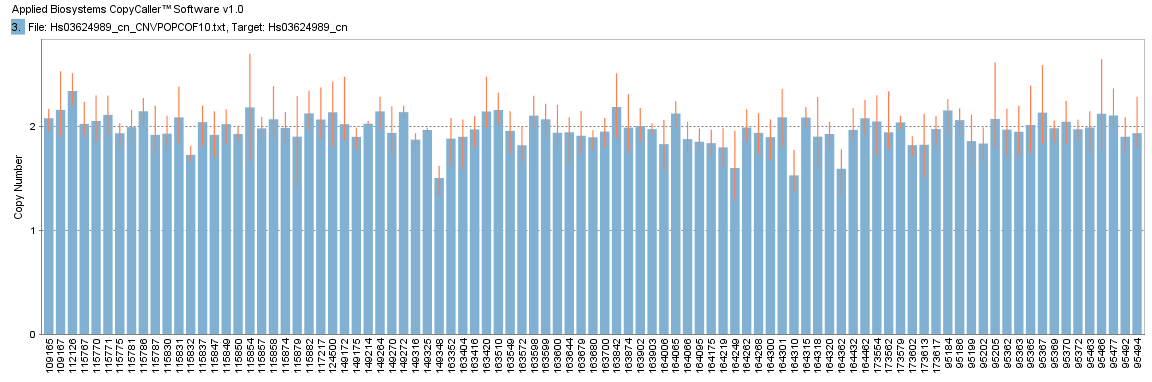

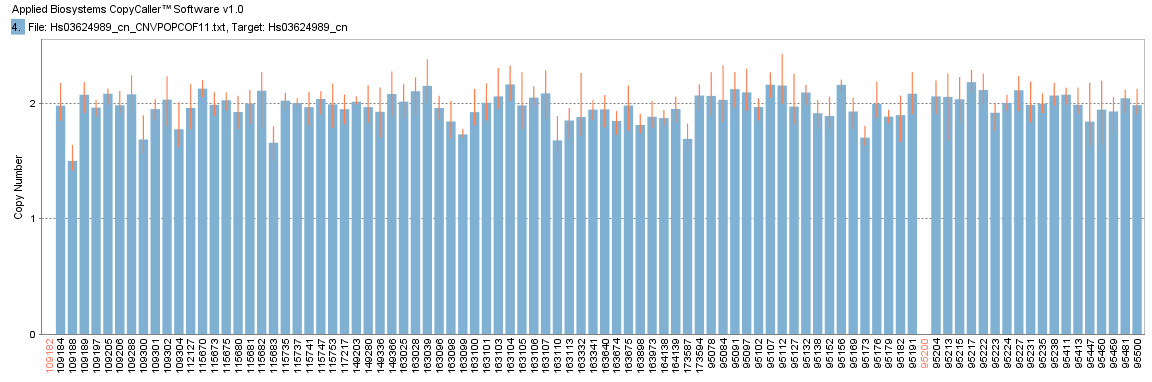


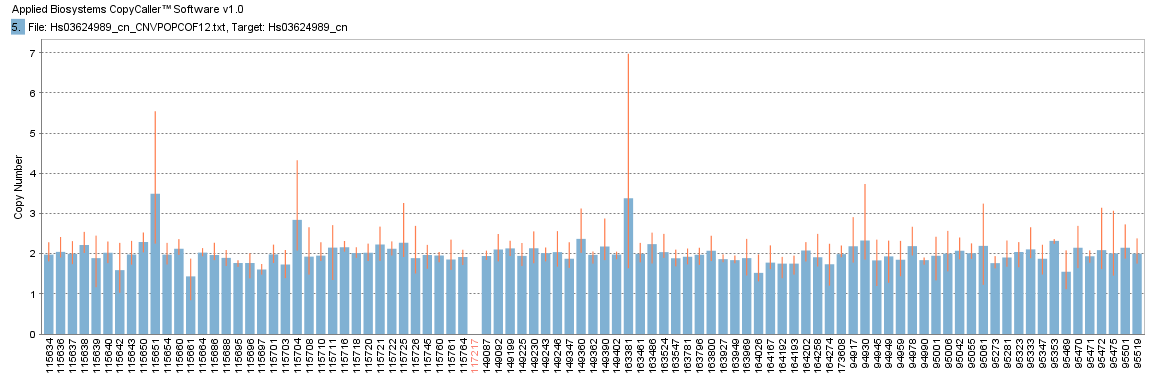

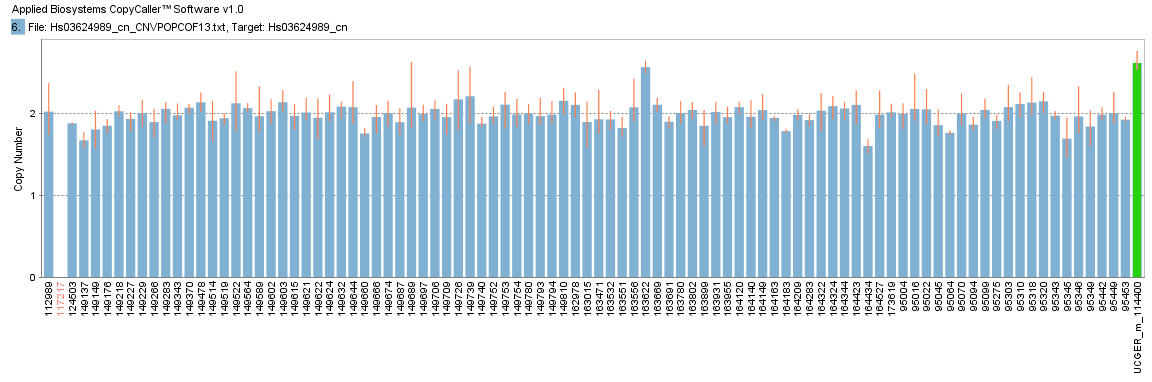


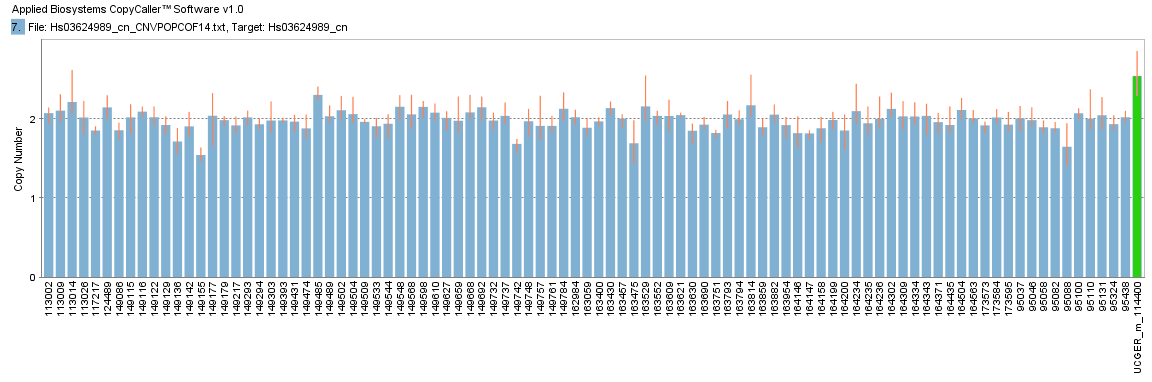

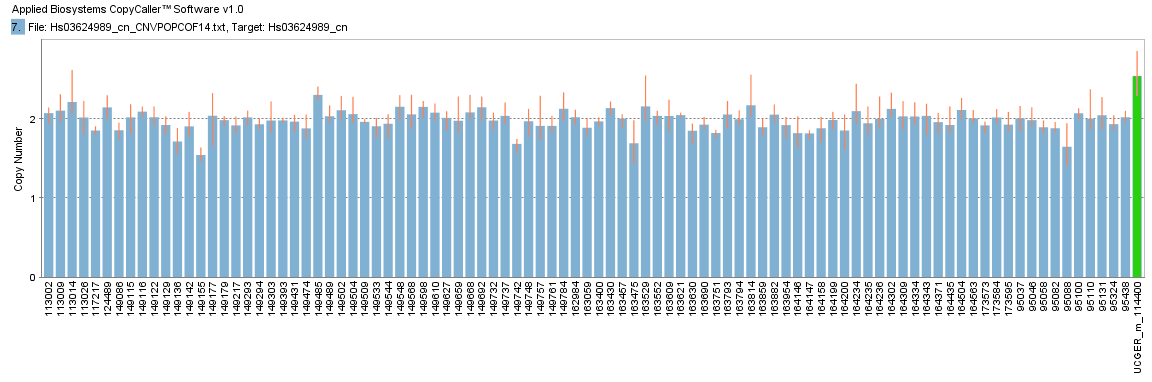


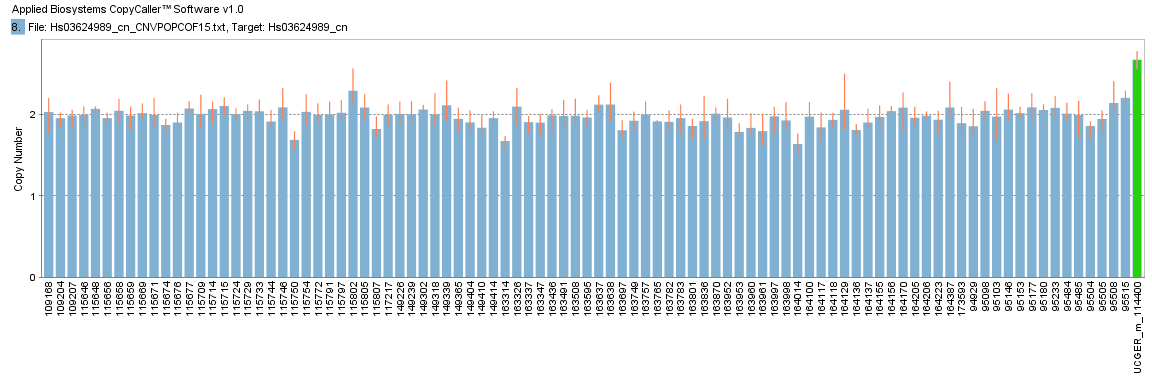

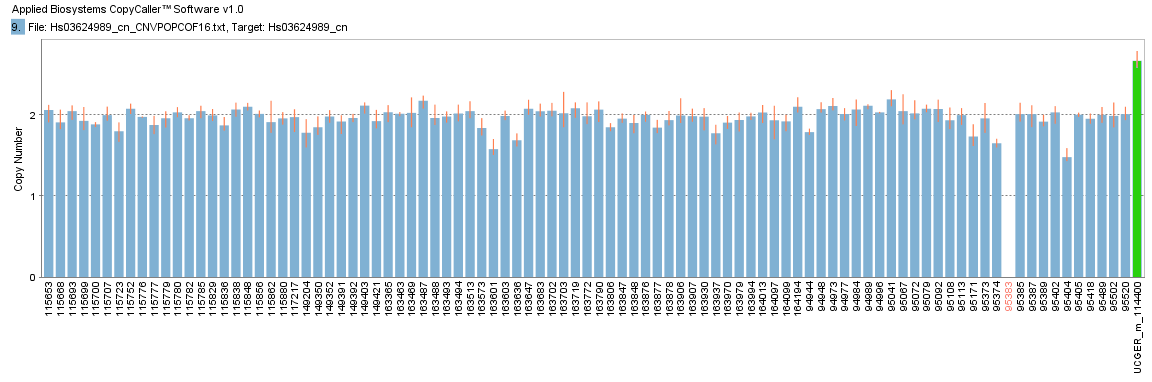


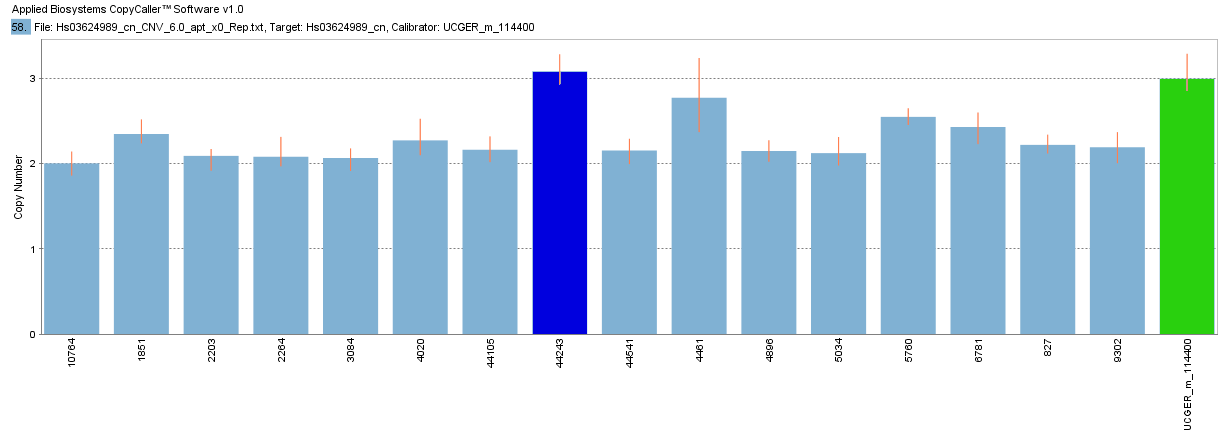


**Suppl. Figure 5. Independent Replication of Duplication 8q24.3 with TaqMan^®^ CNV assays.** Each graph of the figure shows the result of one real time PCR TaqMan^®^ plate, with the samples at the x-axis and the intensities at the y-axis. Graphs with red frame are from case plates and blue frames from control. Duplication carriers are highlighted by dark blue bars. Samples with green bars are calibrator samples, which are samples with confirmed duplication. These calibrator samples are used to identify the CNV carrier within the replication sample set. TaqMan^®^ quality control was based on confidence greater or equal to 94%.


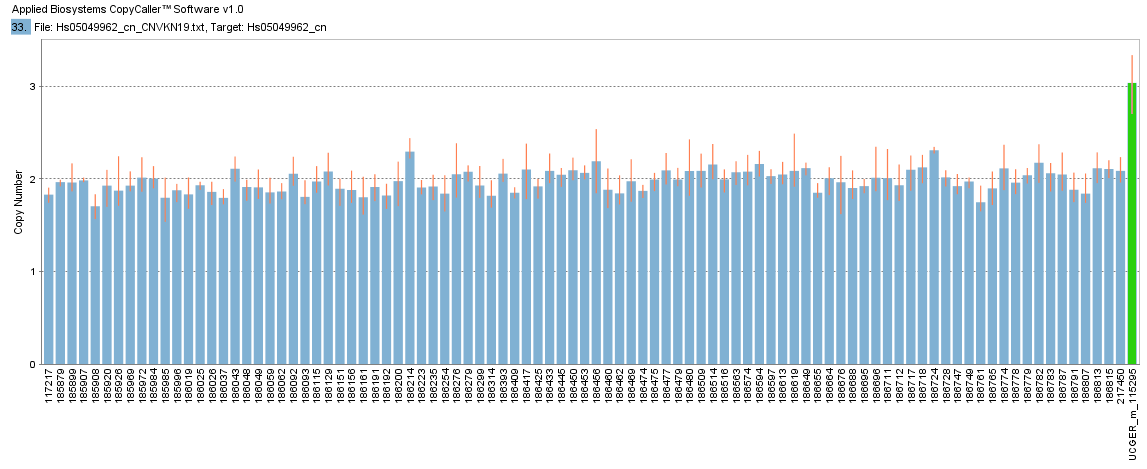

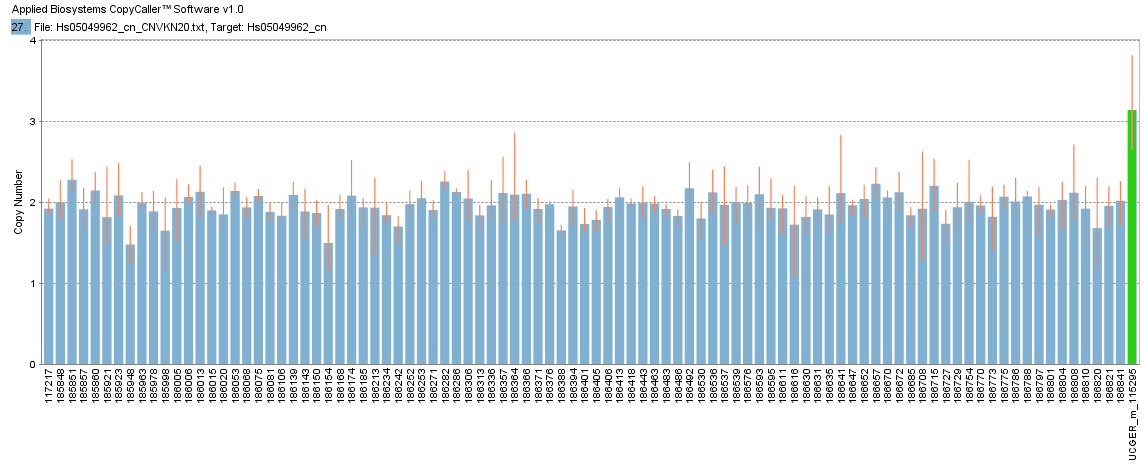


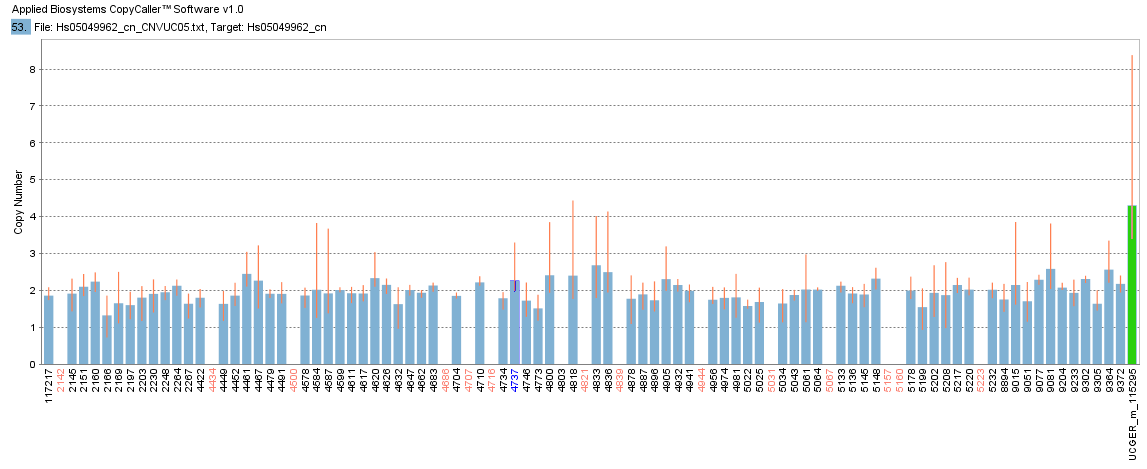

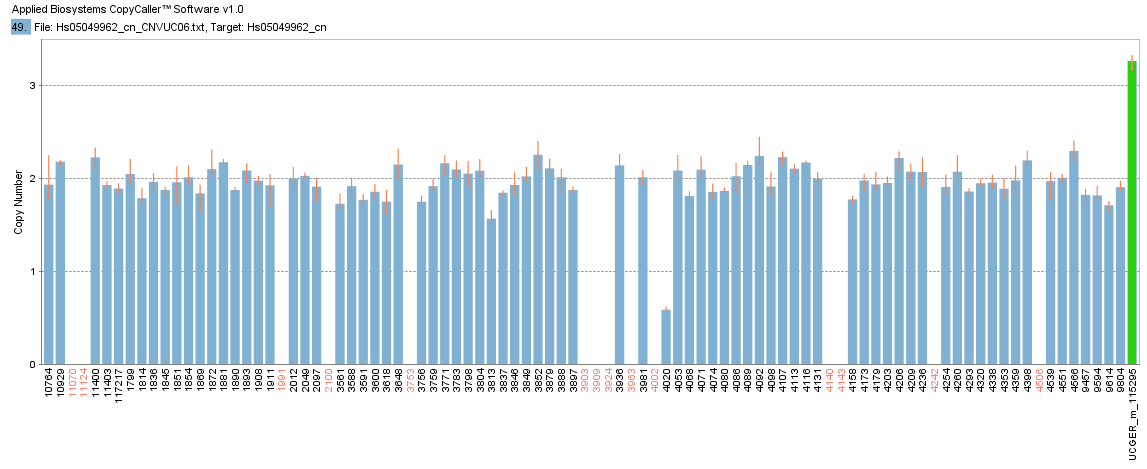


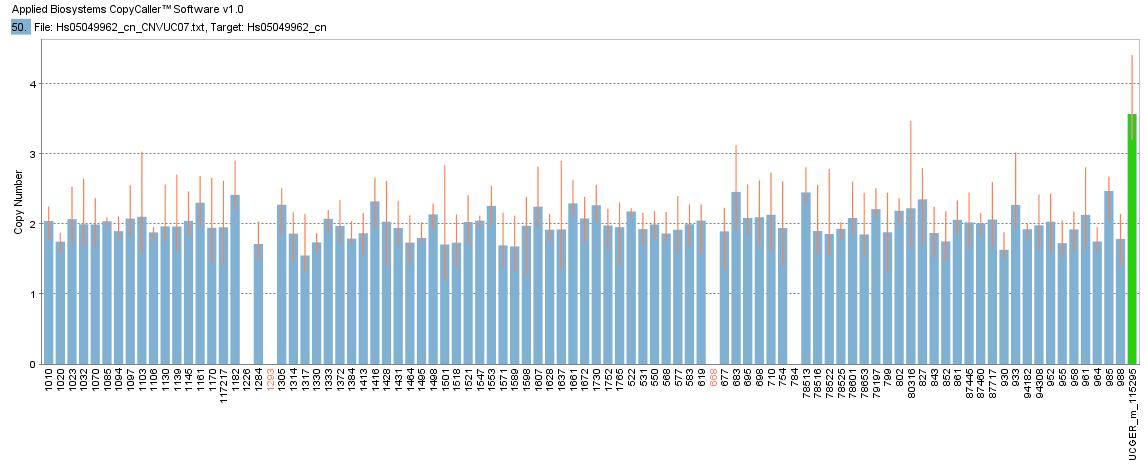

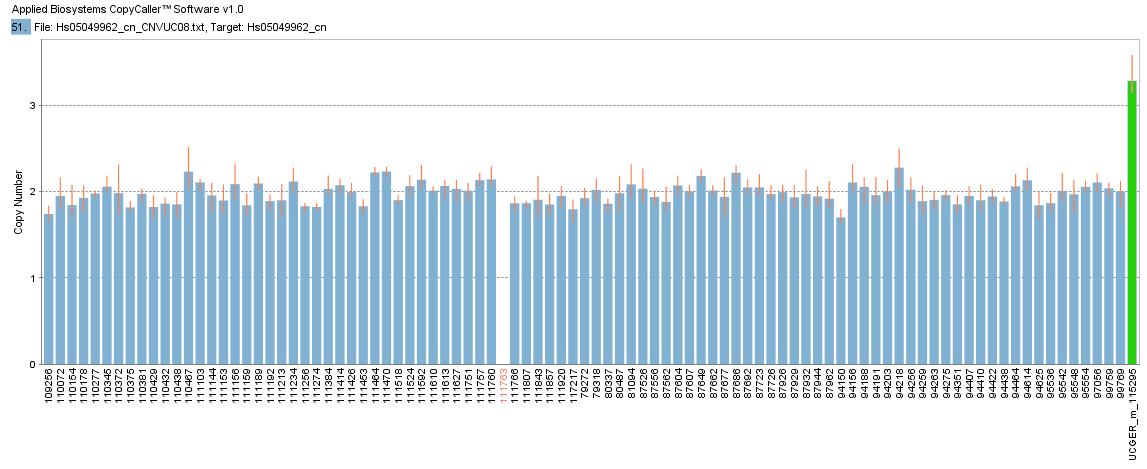


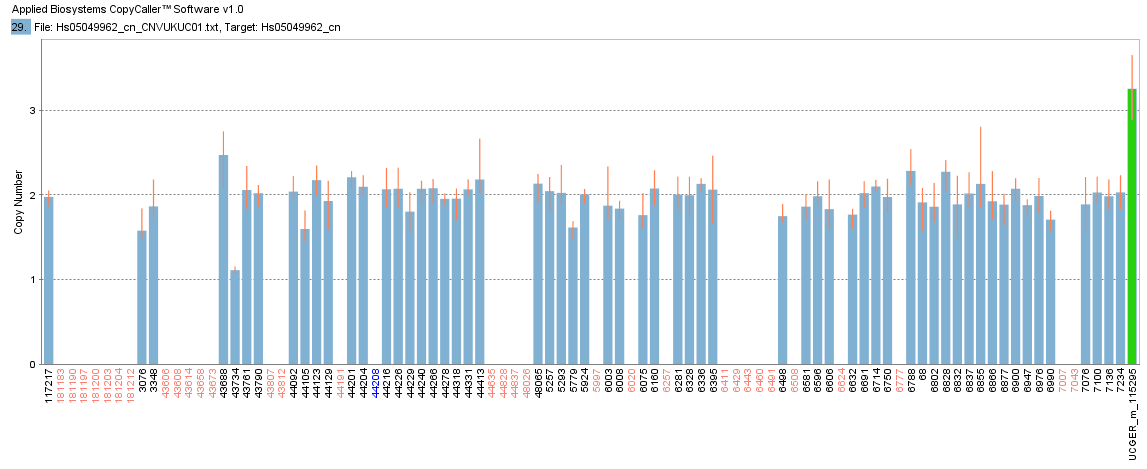

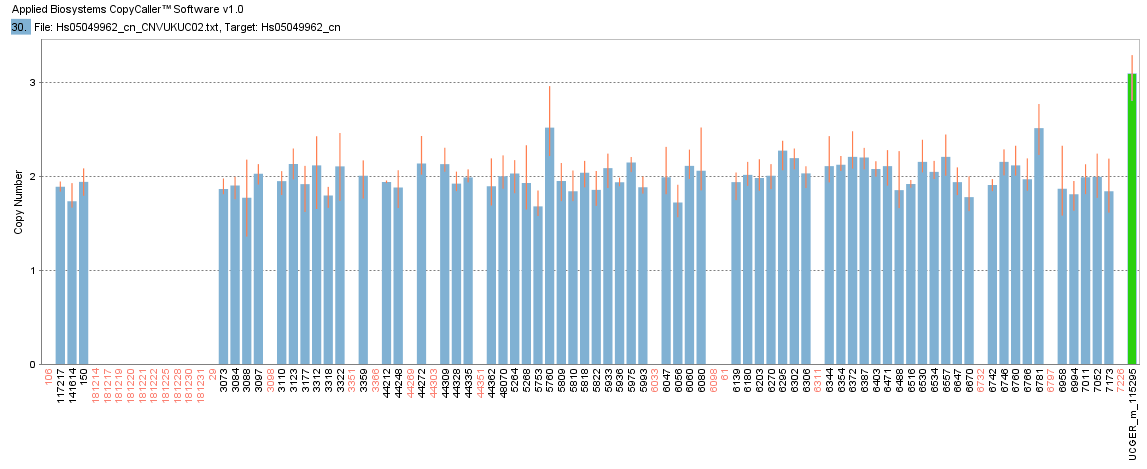


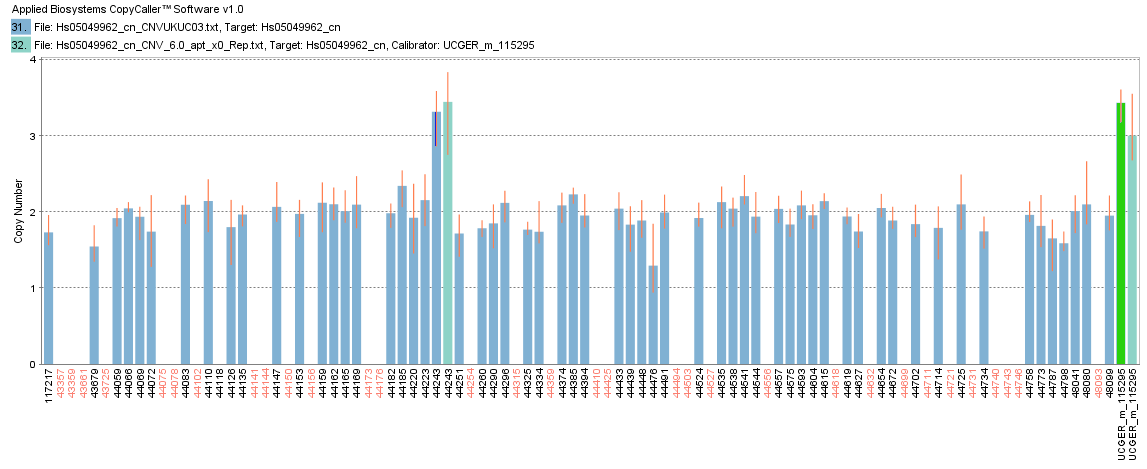


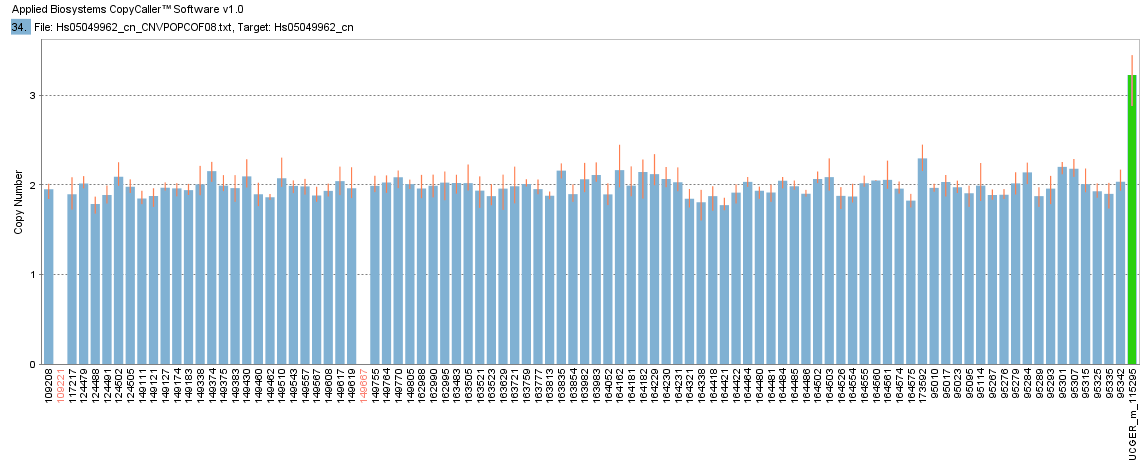

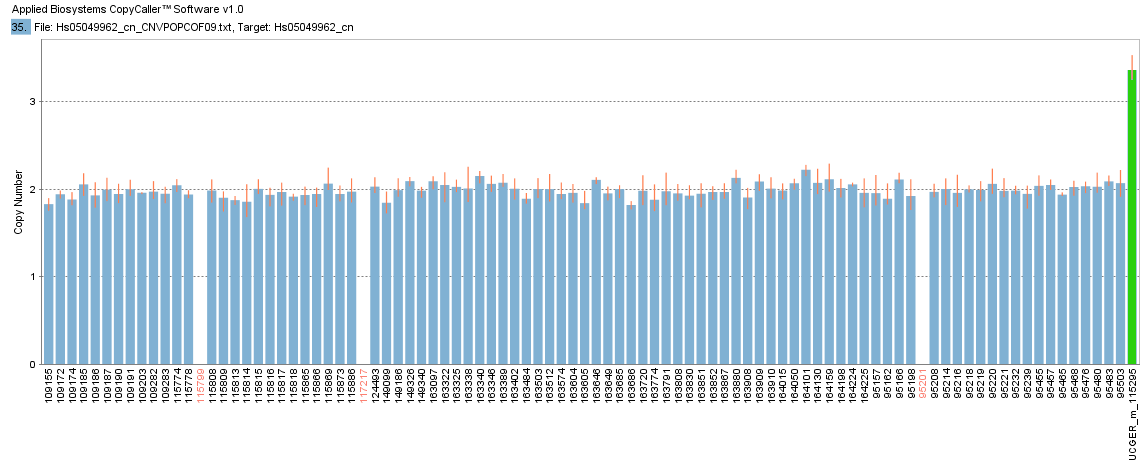


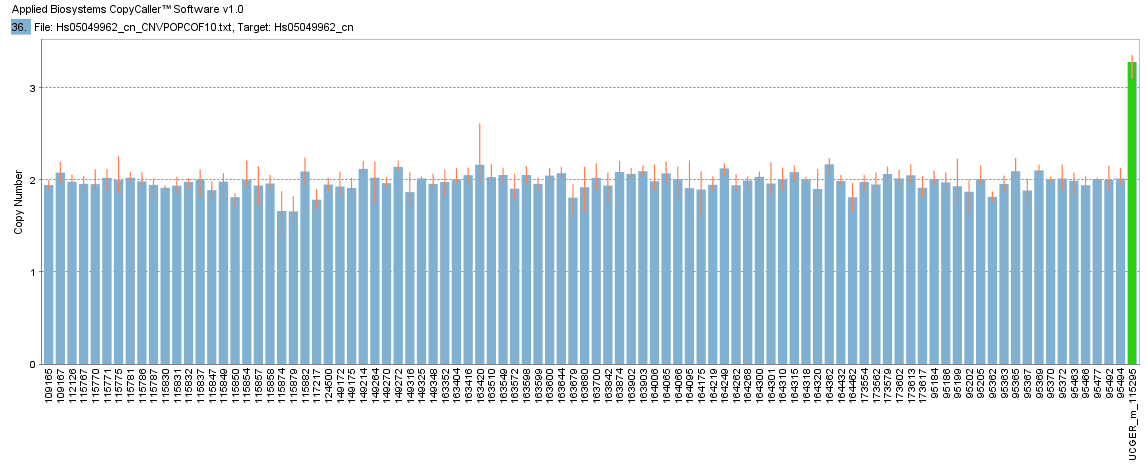

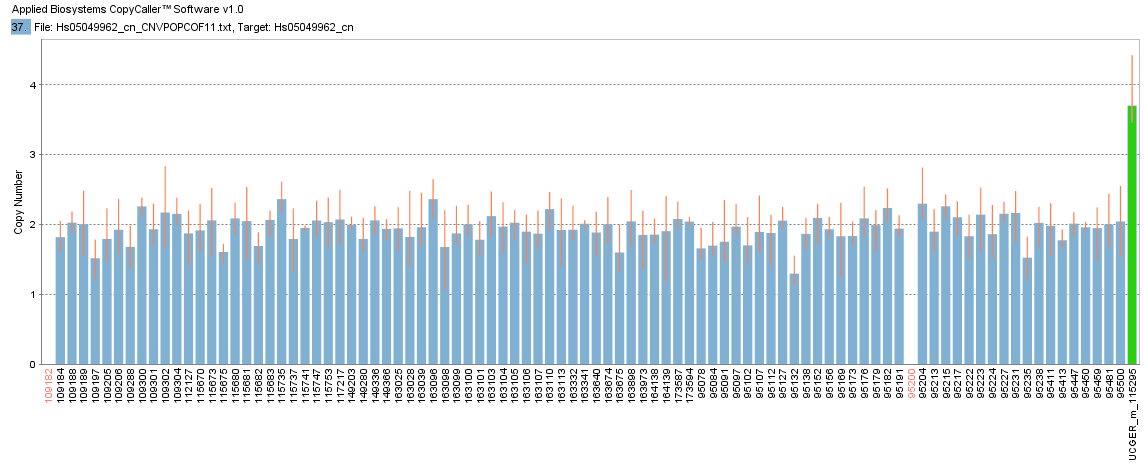


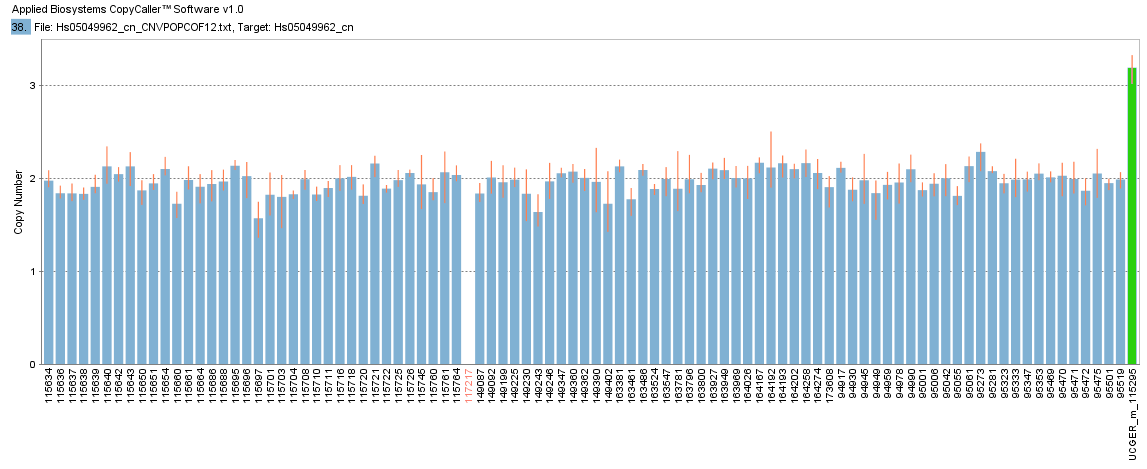


**Suppl. Figure 6. *In Silico* Replication of the 24 (13) rare CNV regions within the Norwegian data set**

24 rare CNVs identified by the initial CNV screening in German discovery (**Suppl. Figure 1**), were evaluated in the Norwegian *in silico* replication data set. Eleven of the 24 regions have no predicted CNVs within the Norwegian sample set (neither cases nor controls) and are not visualized here. Each picture consists of two main panels. The upper panel shows the case CNVs and the lower panel the control CNVs.

**Suppl. Table 2. Description of *in silico* controls.** The three relevant CNVs were evaluated through the SNP array- based genotyping data sets of a total of 6724 individuals recruited in previous studies. The number of controls taken from each study, the sample origin, the array type, the PubMed-id and a short description of the publication are showed in the table. The three right columns show the number of array probes lying within the genomic region of the 3 CNVs which we found in our study. The red highlighted background indicates the sample sets not used for a specific locus(less than 10 probes). The final row shows the number of *in silico* controls we used for each specific locus.

| samples | Ethnicity | Array | | PMID | Description | chr13:94781525-94797285 | chr7:5786323-5905210 | chr8:140390975-140524875 |
| --- | --- | --- | --- | --- | --- | --- | --- | --- |
| 60 | CEU | | Illumina 1M Duo | 18776910 | Hapmap | 9 | 63 | 49 |
| 445 | Caucasian | | Illumina 550K, v3 | 17116639 | NINDS ctrl set from Andy Singleton, II | 5 | 12 | 29 |
| 283 | Caucasian | | Ill. Human Hap300 | 16516587 | CAP subset of PARC (Caucasian only) | 4 | 5 | 16 |
| 653 | Caucasian | | Ill. Human Hap300 | 11434828 | PRINCE subset of PARC (Caucasian only) | 4 | 5 | 16 |
| 231 | Caucasian | | Illumina 610quad | 16516587 | additional CAP samples | 5 | 34 | 28 |
| 551 | Caucasian | | Illumina 610quad | 11434828 | additional PRINCE samples | 5 | 34 | 28 |
| 1320 | various European | | Affymetrix 6.0 | 19592680 | European unrelated from CHOP DB | 13 | 54 | 107 |
| 3181 | various European | | Affymetrix 6.0 | 18668038 | Ctrls. from ISC paper on Schizophrenia | 13 | 54 | 107 |
| 6724 |  | |  |  |  | 5788 controls | 6724 controls | 6724 controls |

**Suppl. Figure 7. Expression analysis of *ABCC4* and *CLDN10* in intestinal biopsies of a UC patient panel** Real-time PCR through pre-designed TaqMan expression assays was carried out for *ABCC4* (A) and *CLDN10* (B) genes. Biopsy samples included one deletion carrier with acute inflammation (1), one deletion carrier without acute inflammation (2), thirty UC patients with acute inflammation and wildtype genotype (3) and thirty cases without acute inflammation and wildtype genotype (4).

| 1 | Del13q32.1 carrier, no inflammation |
| --- | --- |
| 2 | Del13q32.1 carrier, acute inflammation |
| 3 | Non-carrier, inflamed |
| 4 | Non-carrier, not inflamed |

**Suppl. Figure 8. Deletion 13q32.1 in WTCCC2 data set.** *Evaluation* of Del13q32.1 within the UK-WTCCC2 data set is visualized here. The first graph of this figure shows an overview of the CNV prediction for that region (Affymetrix Power Tools copy-number-workflow). The pictures show the raw data visualization of LRR in the top part and B allele frequency (BAF) in the lower part. Non polymorphic probe sets are blue and SNP probesets black. RefGene annotation is added with purple arrows. The red bar between RefSeq annotation and raw data highlights the predicted deletion.

**Suppl. Figure 9. Duplication 7p22.1 in WTCCC2 data set.** The raw data visualization of LRR in the top part and B allele frequency (BAF) in the lower part. Non polymorphic probe sets are blue and SNP probesets black. RefGene annotation is added with purple arrows. The blue bar between RefSeq annotation and raw data highlights the predicted duplication.

**Suppl. Figure 10. Duplication 8q24.3 in WTCCC2 data set.** The pictures show the raw data visualization of LRR in the top part and B allele frequency (BAF) in the lower part. Non polymorphic probe sets are blue and SNP probesets black. RefGene annotation is added with purple arrows. The blue bar between RefSeq annotation and raw data highlights the predicted duplication.

**Suppl. Figure 12. Evaluation of the 24 rare CNV within the UK (WTCCC2) data set.** The pictures of this figure show the regions, identified by the initial CNV screening (**Suppl. Figure 1**), within the UK *in silico* replication sample set. Each picture consists of two main panels. The upper panel shows the case CNVs and the lower panel the control CNVs. Each panel is separated into three subpanels from bottom to top. First in purple arrows the RefSeq genes are annotated. Followed by probe sets with SNP probe sets in black and copy number probesets in blue. The upper subpanel shows the predicted CNVs.

**Suppl. Figure 14. Principle component analysis of the screening panel with the HapMap 3 reference data set.** The first two Principal components were used, with eigenvector 1 at the X-axis and eigenvector 2 at the Y-axis. Each point represents the position of an individual. The screening panel samples are labeled with “IKMB” and/or “IKMB_CASE”,”IKMB_CTRL”, while the remaining samples are 3 letter-labeled by the population identifier. The three letter label code can be translated as follows. African ancestry in Southwest USA(ASW),Utah residents with Northern and Western European ancestry from the CEPH collection(CEU),Han Chinese in Beijing, China(CHB),Chinese in Metropolitan Denver, Colorado(CHD),Gujarati Indians in Houston, Texas(GIH),Japanese in Tokyo, Japan(JPT),Luhya in Webuye, Kenya(LWK),Mexican ancestry in Los Angeles, California(MEX),Maasai in Kinyawa, Kenya(MKK),Toscans in Italy(TSI) and Yoruba in Ibadan, Nigeria(YRI), The smaller plot with the light grey backround at the bottom left corner is a zoom into the CEU/TSI/IKMB region, which has a light grey background in the main plot. Within the smaller plot, all CNV carrier having one of the identified three CNVs of our study are shown as black dots.

**Suppl. Figure 15. Number of copy number segments per sample.** The figure below shows box plots based on the number of segments per sample. A) The number of segments per sample in the UC screening panel. Cases at the left, controls at the right. The y-axis is in log scale. B) The number of segments per sample in the UK UC replication panel. Cases at the left, controls at the right. The y-axis is in log scale.

**References**

1. Lennard-Jones, J.E. Classification of inflammatory bowel disease. *Scand J Gastroenterol Suppl*

170, 2‐6; discussion 16‐9(1989)

2. Wichmann, H.E., Gieger, C. & Illig, T. KORA‐gen‐‐resource for population genetics, controls and a broad spectrum of

disease phenotypes. Gesundheitswesen 67 Suppl 1, S26‐30(2005)

3. Moum B, Vatn MH, Ekbom A, Aadland E, Fausa O, Lygren I, Sauar J, Schulz T, Stray N. Incidence of ulcerative colitis and indeterminate colitis in four counties of southeastern Norway, 1990-93. A prospective population-based study. The Inflammatory Bowel South-Eastern Norway (IBSEN) Study Group of Gastroenterologists. Scand J Gastroenterol. 1996;31(4):362-6.

4. Browning BL, Yu Z (2009) Simultaneous genotype calling and haplotype phasing improves genotype accuracy and reduces false-positive associations for genome-wide association studies. Am J Hum Genet 85: 847-861.

5. Price AL, Patterson NJ, Plenge RM, Weinblatt ME, Shadick NA, et al. (2006) Principal components analysis corrects for stratification in genome-wide association studies. Nat Genet 38: 904-909.

6. Wittig M. *et al.* CNVineta: a data mining tool for large case-control copy number variation datasets. *Bioinformatics*. **26**:2208-9 (2010)
